# A single-cell transcriptomic atlas reveals the cell differentiation trajectory and the response to virus invasion in swelling clove of garlic

**DOI:** 10.1093/hr/uhae365

**Published:** 2025-01-03

**Authors:** Song Gao, Fu Li, Zheng Zeng, Qiaoyun He, Hassan H A Mostafa, Suling Zhang, Taotao Wang, Yanzhou Wang, Touming Liu

**Affiliations:** Key Laboratory of Biobreeding for Specialty Horticultural Crops of Jiangsu Province, College of Horticulture and Landscape Architecture, Yangzhou University, No. 88, Southern road of Daxue, 225009, Yangzhou, China; Key Laboratory of Biobreeding for Specialty Horticultural Crops of Jiangsu Province, College of Horticulture and Landscape Architecture, Yangzhou University, No. 88, Southern road of Daxue, 225009, Yangzhou, China; Institute of Bast Fiber Crops, Chinese Academy of Agricultural Sciences, No. 348, Western road of Xiajiahu, 410205, Changsha, China; Institute of Bast Fiber Crops, Chinese Academy of Agricultural Sciences, No. 348, Western road of Xiajiahu, 410205, Changsha, China; Central Laboratory of Organic Agriculture, Agricultural Research Center, 9, Cairo Univ. st., 12556, Giza, Egypt; Shanghai OE Biotech. Co., Ltd, No. 1188, Road of Lianhang, 201100, Shanghai, China; Shandong Dongyun Research Center of Garlic Engineering, No. 52, Jinze Road, Yushan street, 272200， JinXiang, China; Institute of Bast Fiber Crops, Chinese Academy of Agricultural Sciences, No. 348, Western road of Xiajiahu, 410205, Changsha, China; Shandong Dongyun Research Center of Garlic Engineering, No. 52, Jinze Road, Yushan street, 272200， JinXiang, China; Key Laboratory of Biobreeding for Specialty Horticultural Crops of Jiangsu Province, College of Horticulture and Landscape Architecture, Yangzhou University, No. 88, Southern road of Daxue, 225009, Yangzhou, China; Shandong Dongyun Research Center of Garlic Engineering, No. 52, Jinze Road, Yushan street, 272200， JinXiang, China

## Abstract

The garlic bulb comprises several cloves, the swelling growth of which is significantly hindered by the accumulation of viruses. Herein, we describe a single-cell transcriptomic atlas of swelling cloves with virus accumulation, which comprised 19 681 high-quality cells representing 11 distinct cell clusters. Cells of two clusters, clusters 7 (C7) and 11 (C11), were inferred to be from the meristem. Cell trajectory analysis suggested the differentiation of clove cells to start from the meristem cells, along two pseudo-time paths. Investigation into the cell-specific activity of invasive viruses demonstrated that garlic virus genes showed relatively low-expression activity in cells of the clove meristem. There were 2060 garlic genes co-expressed with virus genes, many of which showed an association with the defense response. Five glutathione synthase/reductase genes co-expressed with virus genes displayed up-regulated expression, and the glutathione and related metabolites level showed an alteration in virus-invasive garlic clove, implying the role of glutathione in viral immunity of garlic. Our study offers valuable insights into the clove organogenesis and interaction between garlic and virus at single-cell resolution.

## Introduction

Garlic bulb is the economically important organ in garlic production; it typically consists of several cloves, each of which comprises one protective leaf and one storage leaf, which undergo rapid enlargement during the early stage of clove development. However, in the late stage of clove swelling growth, the nutritive components of the protective leaf transfer into the storage leaf, causing the protective leaf to become a thin flake covering the storage leaf [1]. Storage organs resulting from the swelling growth of vegetative organs are widespread in the plant kingdom (e.g. potato tubers). Cellular observations suggest that increased cell number and size of parenchymal cells in the cortex, pith, and circummedullary tissues are major reasons for tuber swelling in potatoes [2]. In onion, the expansion of cells in leaf sheath is deemed to be a morphological basis underlying bulb formation [3]. Recent spatial RNA-seq suggests that spongy mesophyll cells are the dominant cell type in bulbs, and proposes that cortical microtubule rearrangement might be pivotal for bulb swelling growth [4]. Notably, swelling of potato tubers and onion bulbs has been linked to the control of *Flowering Locus T*-like genes [5, 6], indicating a potential overlap in their swelling mechanism. Although some candidates associated with swelling growth of garlic clove have been identified based on genetic and genomic analyses [7–10], current understandings of clove growth remain limited.

Owing to the sterility of cultivars, garlic reproduction relies on vegetative organ cloves, leading to significant virus accumulations in individual garlic plants. At least 20 virus types have been identified in garlic, including aphid-borne potyviruses (e.g. onion yellow dwarf and leek yellow stripe viruses), carlaviruses (e.g. garlic common latent and shallot latent viruses), and mite-borne allexiviruses (including garlic viruses A, B, C, and D) [11–19]. Virus accumulation severely impacts garlic growth. Long-term observations over 5 years have shown that viral accumulation markedly reduces bulb yield and that viral damage increases significantly with the number of planting years [20]. Consequently, viruses have become some of the most common pathogens, causing significant bulb yield losses in garlic crops worldwide. However, the mechanism underlying the impact of viruses on bulb growth remains largely unknown.

Plant viruses are typically acellular organisms and minimally consist of nucleic acids encapsulated in a capsid or coat protein. Due to the highly limited number of biological functions encoded by the viral genome, plant viruses rely on the intracellular machinery of the host for the replication of their genomes, the expression of viral genes, and infection establishment [21]. Thus, plant viruses are specific pathogens that are active only inside the host cell [22]. Unlike the fungal and bacterial pathogens, plant viruses generally try to keep their hosts alive for as long as possible, which makes the virus–plant interaction more prolonged. Because of the acellular characteristics of viruses, the virus–plant interaction often highly depends on the internal pathways of the host [23]. Therefore, the invasion and interaction of viruses with host plants have distinct differences from that of fungal and bacterial pathogens. For instance, viral invasion of host plants shows cell-specific differences, and stem cells can be protected against viral intrusion through a conserved strategy based on *WUSCHEL*-mediated antiviral immunity in plants [24].

Single-cell RNA sequencing (scRNA-seq) technology has been proven to be a powerful tool for identifying cell-specifically expressed genes and exploring their roles in finely controlled plant–pathogen interaction. Recent scRNA-seq studies uncover the signal pathways that plants use to adapt to the invasion of fungi, including *Fusarium verticillioides* [25], *Colletotrichum higginsianum* [26], and powdery mildew [27]. The scRNA-seq of *Arabidopsis* exposed to the bacteria *Pseudomonas syringae* leads to the identification of distinct pathogen-responsive cell clusters exhibiting transcriptional responses ranging from immunity to susceptibility [28]. Unlike the pathogen of bacteria and fungus, viruses are active only inside the host cell. A recent study performed an scRNA-seq of soybean infected by soybean mosaic virus, and identified two glutathione S-transferase genes, which were induced expression by virus infection, representing an important insight into the interaction between virus and host plant at single cell level [29]. In garlic, although clonal propagation causes great accumulation of virus, the active characteristics of viruses in garlic cells remain rarely known. Herein, to overview the overall landscape of viruses in garlic clove at single cell resolution, we performed an scRNA-seq analysis using three samples representing typical stages of clove swelling growth. Then, cells in swelling cloves were identified, and the expression of garlic and virus genes and the interaction of garlic and virus were characterized in these cells. This study provides important insights into garlic–virus interaction, and is valuable for guiding efforts to reduce garlic viruses in garlic cultivation.

## Results

### A single-cell transcriptomic atlas of swelling cloves

**Figure 1:**
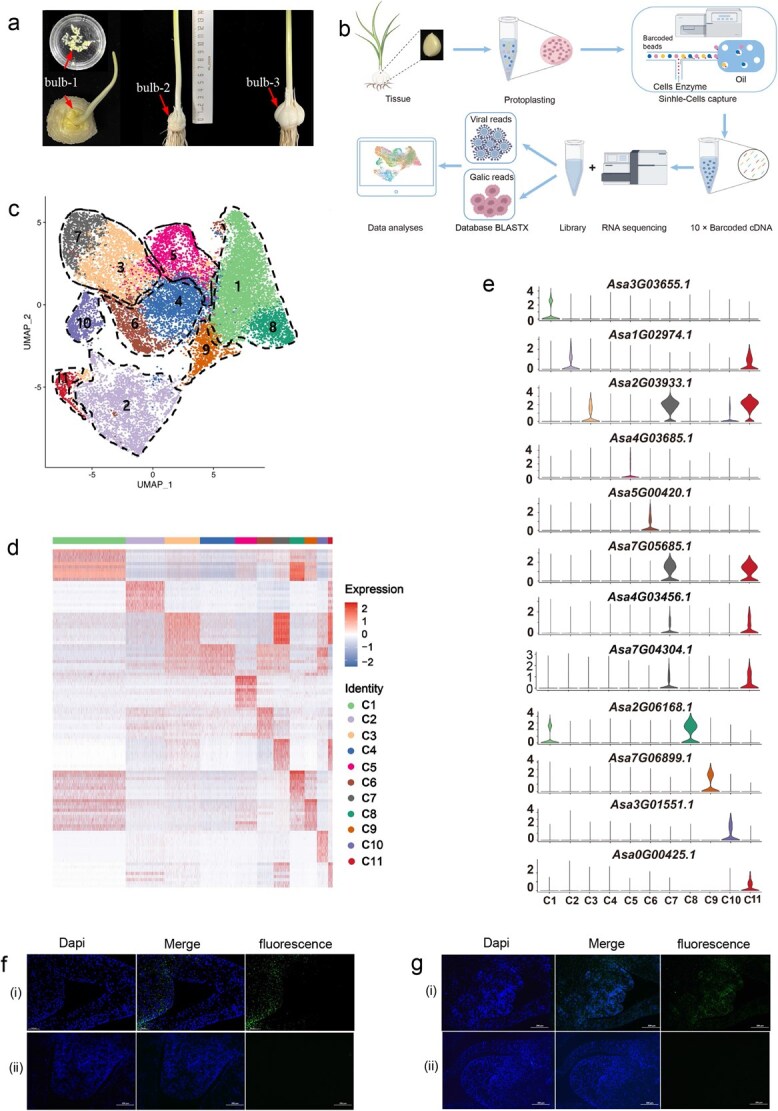
Single-cell RNA sequencing (scRNA-seq) for cloves under swelling growth. (**a)** Cloves under three different developmental stages were collected for scRNA-seq analysis. (**b)** A schematic diagram of workflow for single-cell RNA sequencing of garlic cloves. (**c**) UMAP visualization of 11 clusters derived from 19 681 high-quality cells filtered from three clove samples. Parenchyma and meristematic cell types are indicated in the bottom right corner of UMAP. (**d)** A heatmap showing the expression of the top 10 marker genes in each cell cluster. (**e)** Violin plots showing the expression of representative marker genes in each cell cluster. RNA fluorescence *in situ* hybridization assay for (**f**) *Asa4G03456.1* and (**g**) *Asa7G05685.1* in bulb-1 using the (i) antisense and (ii) sense probes labeled by digoxigenin, respectively. A blue signal indicates the cell nucleus stained by the 4′,6-diamidino-2-phenylindole (Dapi) solution, and a green fluorescence signal represents the signal of the digoxigenin-labeled probe

To obtain a relatively comprehensive single-cell transcriptomic atlas, we selected clove samples (bulb-1, bulb-2, and bulb-3) collected from three typical clove growth stages for scRNA-seq analysis. Bulb-1 cloves were young and were beginning to swell; bulb-2 cloves were actively swelling; and bulb-3 cloves had completed enlargement and were filling (Fig. 1a). Next, we performed an scRNA-seq analysis for these samples according to the workflow shown in Fig. 1b. After sequencing individually for cloves of three stages, approximately 4.92 billion scRNA-seq reads were analyzed, and the identified cell count ranged from 6188 to 10 568 in the three samples (Supplementary Table 1). After stringent quality filtering, a total of 19 681 high-quality cells remained among the three clove types. These cells were clustered into 11 distinct cell groups using the *FindClusters* function of Seurat software (Fig. 1c) [30]. The median gene and UMI number per cell were 1667 and 2907 in bulb-1, 1405 and 2451 in bulb-2, and 727 and 936 in bulb-3, respectively (Supplementary Table 1). There were distinct differences in cell number and expressed genes in each cell type. For example, cluster 1 (C1; ‘cluster’ of cloves is hereafter abbreviated as C) was the largest group, with 5130 cells and 29 002 expressed genes, and C11 only comprised 327 detected cells (Supplementary Fig. 1). Of these clove cell-expressed genes, 3036 had relatively high relative expression in several of the 11 cell clusters (Fig. 1d; Supplementary Data 1; Supplementary Fig. 2). C3 (26.03%), C2 (30.68%), and C1 (58.91%) were the most prevalent cell types in bulb-1, bulb-2, and bulb-3, respectively (Supplementary Table 2). Overall, these results indicated considerable diversity in cell types in the swelling cloves.

### Identifying cells of the clove meristem

There were two MADS-box transcription factor-encoding genes *Asa4G03456.1* and *Asa7G04304.1* that expressed specifically in C7/C11 cells (Fig. 1e). MADS-box genes play pivotal roles in regulating meristem cell differentiation and determining meristem cell fate in plants [31, 32], and numerous MADS-box genes have been identified to express specifically in apical meristem and/or bud primordia [31–35]. Therefore, we performed an RNA fluorescence *in situ* hybridization (FISH) assay in bulb-1 using the digoxigenin-labelled antisense probe of *Asa4G03456.1* cDNA, and the sense probe was used as a parallel control. The result detected strong fluorescence signals in the apical meristem of the clove (Fig. 1f), indicating that C7 and C11 cells were from clove meristem.

Interestingly, of the top 100 highly expressed genes in C7 and C11 cells, 99 and 97 functioned, respectively, as putative histone-encoding genes (Supplementary Data 2), many of which displayed specific expressions in these two clusters (Fig. 1e). Enrichment analysis of genes expressed in cells of these two clusters revealed that they were significantly enriched in gene ontology (GO) terms associated with nucleosomes, such as ‘nucleosome’ and ‘nucleosome assembly’ (Supplementary Fig. 3). Histone protein synthesis is tightly coupled to DNA replication owing to the roles of these proteins in packing the newly replicated DNA into chromatin, and histone gene expression is cell-cycle-regulated, with a 35-fold increase in the transcript abundance when cells enter the stationary phase [36]. Previous *in situ* hybridization and GUS reporter expression assays indicate that histone genes are expressed in meristematic cells [37–40]. These histones were used in the present study to identify their orthologs in the garlic genome (Supplementary Table 3), and their expressed cells were then detected, revealing that these orthologous genes were expressed in C7 and C11 cells (Supplementary Fig. 4). Furthermore, we synthesized the digoxigenin-labelled antisense probe of *Asa7G05685.1* that was one of these C7/C11 cell-expressed-specifically histone genes (Fig. 1e), and this probe then was used for RNA *in situ* hybridization. The result showed strong fluorescence signals at the apical meristem of the young clove, with a distribution of scattered manner across the apical meristem (Fig. 1g), which is consistent with that of histone *H2A* of tomato [37], further verifying that C7 and C11 cells were from clove meristem. The high expression of numerous histone genes in these cells may be for meeting the demand to pack the DNA replicated from the giant garlic genome (~16.9 Gb) during meristem cell division.

### Cell trajectory of swelling clove

**Figure 2:**
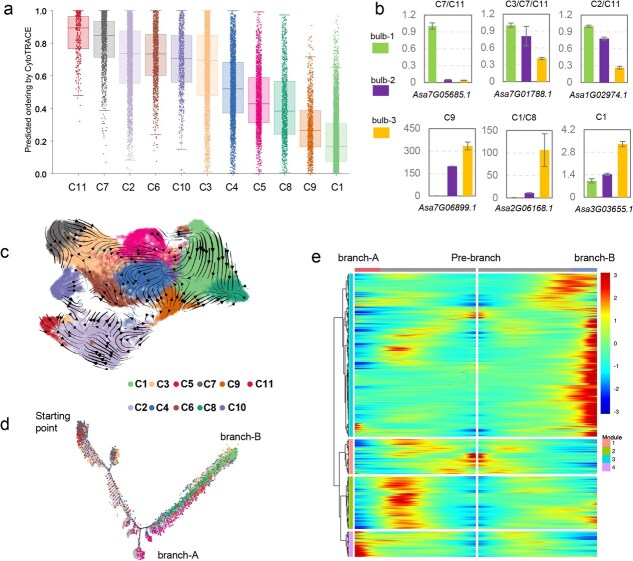
Cell differentiated trajectory of swelling clove. **(a)** Differentiation states of cells in each cluster predicted by CytoTRACE. (**b**) Relatively expressed levels of six cell-expressed-specifically genes in three bulb samples from the analysis of Real-time fluorescence quantitative PCR. The word above each histogram indicates the cell cluster expressed specifically for corresponding gene. (**c**) RNA velocity map of cells from 11 cluster. The direction of the arrow represents the direction of cell development. (**d**) Trajectory of clove cells along the pseudo-time path. Each dot represents a single cell. Two branches (Branch-A and Branch-B) are shown. (**e**) Heatmap showing the expression of the branch-dependent genes along the pseudo-time. The branch point (shown in the middle of heatmap; grey line) is the beginning of pseudo-time. Left (Branch-A) and right (Branch-B) sides of heatmap are the end of pseudo-time

To determine the starting and terminal point of cell differentiation, we evaluated the cell differential state by estimating the cyto-TRACE value of cells in each cluster. The results showed that cells from C7 and C11 had the largest cyto-TRACE value (>0.8), whereas those of C1 and C9 were the smallest (<0.3) (Fig. 2a), indicating that cells of meristem stem (C7 and C11) had the largest differential potency, and those of C1 and C9 were the smallest in the differential capacity. Of three investigated clove samples, young bulb-1 cloves were beginning to swell, whereas bulb-3 cloves had completed enlargement and were filling, suggesting cells of bulb-1 possessed larger differential potency than those of bulb-3. We performed a real-time fluorescent quantitative PCR analysis (qRT-PCR) using cell-expressed-specifically genes. The results revealed that three C7 and/or C11 cell-expressed-specifically genes, *Asa7G05685.1*, *Asa7G01788.1*, and *Asa1G02974.1*, displayed the highest expression level in young bulb-1, whereas C1 or C9 cell-expressed-specifically genes, *Asa7G06899.1*, *Asa2G06168.1*, and *Asa3G03655.1*, showed the most transcript abundance in bulb-3 (Fig. 2b), further validating that meristem cells are more popular in differential potency-high bulb-1 than in bulb-3, and those of C1/C9 are more prevalent in bulb-3 than bulb-1. Collectively, our results suggest that the differentiation of clove cells starts from meristem stem (C7 and C11), and terminates to cells of C1 and C9.

**Figure 3:**
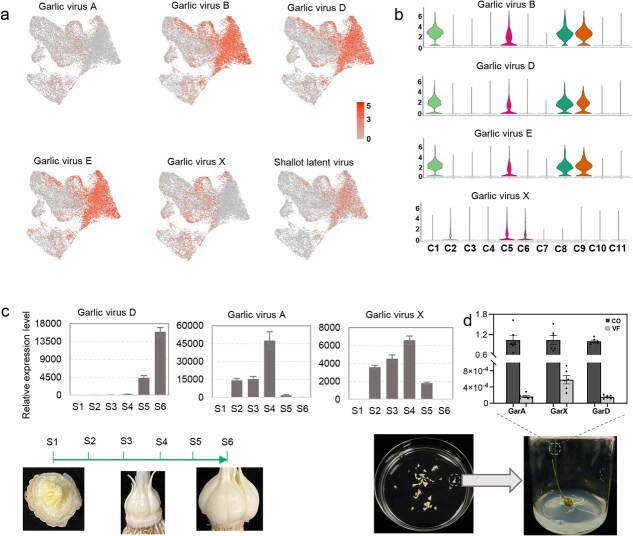
Cell-specific expression of garlic virus genes. (**a)** UMAP shows the expression of genes from six viruses in swelling cloves. *GarVAgp6*, *QY65.gp6*, *X660.gp6*, *GvEgp8*, *GarVXgp6*, and *GarLVgp6* were used to estimate the activity of garlic viruses A, B, D, E, and X, and the shallot latent virus, respectively. (**b)** Violin plots showed the expression of virus genes *X660-GP6* (Garlic viruses D), *GvEgp8* (Garlic viruses E), *GarVXgp6* (Garlic viruses X), and *QY65-gp6* (Garlic viruses B). (**c**), Relative expression level for genes of garlic virus A (*GarVAgp6*), D (*X660.gp6*), and X (*GarVXgp6*) in six stages of clove swelling growth. The bulb figures show the state of clove growth at the beginning (S1) and finishing sampling (S6). (**d**) Relatively expressed levels of *GarVAgp6* (GarA), *X660.gp6* (GarD), and *GarVXgp6* (GarX) in virus-accumulated (CO) and virus-free garlic. The virus-free garlic used is produced by a culture *in vitro* of young cloves. The *y* axis indicates a relative expression level.

We next estimated the cell differential direction of clove cells, as cells of meristem cells as a start point, using an RNA velocity analysis. The results revealed that meristem cells developed toward two directions, namely, C7 and C11 cells differentiated as C3 and C2 types, respectively, and then C2/C3 cells developed as other cell types (Fig. 2c). Further pseudo-time analyses using the cells of C7 and C11 as the starting point in cell differentiation verified two paths for the cell differentiation in the swelling clove (Fig. 2d). Most cells in one path were from C2, C5, and C6 (designated as ‘branch-A’), whereas most of C1, C8, and C9 cells were as another path (designated as ‘branch-B’; Supplementary Fig. 5). We also analyzed the expression level of genes in cells of branch-A and branch-B, compared to the pre-branched cells, resulting in an identification of four expressed modules (Fig. 2e). Genes in module 1 displayed a downregulated expression in cells of both branches, and were enriched in the GO terms associated with genome structure and regulation, such as ‘nucleosome’ and ‘DNA binding’ (Supplementary Fig. 6). Additionally, genes in module 3 showed upregulated expression in the cells of branch-B, and they displayed a significant enrichment in GO terms including ‘nucleus’ and ‘ATP binding’, whereas genes in module 4 displayed upregulated expression in the cells of branch-A, and were enriched in the ‘response to chitin’ and ‘ethylene-activated signaling pathway’ (Supplementary Fig. 6). These results implied that, with the differentiation of cells toward two directions, their functions demonstrated a distinct divergence.

### Cell-specific activity of accumulated viruses in cloves

Garlic production relies on vegetative propagation, resulting in virus accumulation. The single-cell transcriptomic atlas of swelling cloves enables observation of virus activity in cloves at single-cell resolution. We utilized BLASTX to analyze the scRNA-seq reads of three investigated cloves against protein sequences from the Non-Redundant Protein Sequence (NR) Database to identify viral reads. In total, we detected 44.9 million reads from 195 known viruses (Supplementary Data 3). Expression analysis of virus genes using these scRNA-seq reads indicated that garlic viruses A, B, D, E, and X and the shallot latent virus were prevalent in the investigated cloves, especially garlic viruses B, D, E, and X, whose genes showed the highest expression level among various detected viruses (Fig. 3a; Supplementary Fig. 7). Based on the expression level of the genes of each virus, we estimated the activity of the corresponding virus in various clove cell types. Investigation of cell-specific expression for four viruses with the highest activity in investigated cloves revealed that virus genes showed relatively low expression in meristem cells (C7 and C11) and C3, C4, and C10 cells; genes of garlic virus B, D, and E displayed high expression in C1, C8, and C9 cells, while the garlic virus X gene exhibited relatively high expression in C2, C5, and C6 cells (Fig. 3b). These findings suggested distinct viral activity features in the clove cells of the investigated samples.

We compared the activity of garlic viruses in three investigated samples based on the expression level of their genes. The result revealed that virus genes had scarce expression in cells of young clove—bulb-1, and those of four viruses (garlic viruses B, D, E, and shallot latent virus) showed considerable activity in clove cells of bulb-2 and bulb-3 (Supplementary Table 4 and Fig. 8). The gene activity of one of these four viruses, garlic virus D, was chosen randomly for further validation by real-time fluorescent quantitative PCR (qRT-PCR) analysis, and the result showed that, with the swelling and growth of cloves, the transcript abundance of its gene displayed a continuous increase (Fig. 3c). Interestingly, unlike the viruses mentioned above, the genes of garlic viruses A and X became inactive and scarcely appeared in the cells of the matured clove bulb-3 (Supplementary Table 4 and Fig. 8), which was further verified by qRT-PCR analysis (Fig. 3c). These findings suggested two distinct patterns of viral activity in the clove cells. Accordingly, we developed a method for culture young cloves *in vitro* (Supplementary Fig. 9), and the expression of viruses was significantly lower in leaves of *in vitro* cultured garlic than in those of control (Fig. 3d), further validating that limited viruses appeared in cells of young cloves. Because of the relatively high percent of meristem cells in young cloves (Supplementary Table 2), these findings from expression analysis and culture *in vitro* of young cloves further support the conclusion that garlic viruses possess limited activity in meristem cells.

### Gene expression in response to a viral infection

**Figure 4:**
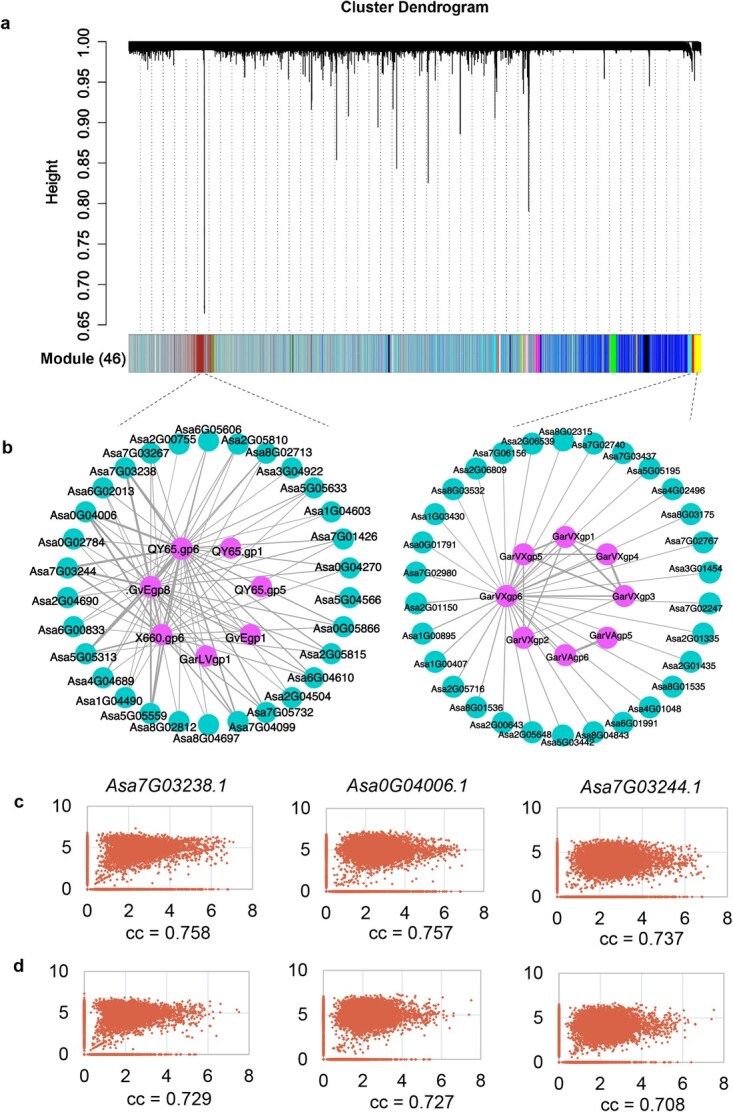
Co-expression of garlic and virus genes. (**a)** Cluster dendrogram of garlic and virus genes from the weighted gene co-expression network analysis. A total of 46 modules were identified and are indicated by different colors in the box below. (**b**) Top 30 garlic genes with correlations with virus genes in the expression of two co-expressed modules. Outer and inside circles indicate garlic and virus genes, respectively. (**c)** Correlation coefficient (cc) between the FPKM value of three garlic genes and *QY65.gp6* of garlic virus B in 19 681 clove cells. (**d)** Correlation coefficient between the FPKM value of three garlic genes and *GvEgp8* of garlic virus E in 19 681 clove cells

The co-expression of garlic and virus genes in 19 681 clove cells was investigated, resulting in the identification of 46 co-expressed modules (Fig. 4a). Genes of garlic viruses B, D, and E and the shallot latent virus co-expressed with 1378 garlic genes were grouped in one module (brown), whereas genes of garlic viruses A and X were identified in another co-expressed module along with 682 garlic genes (yellow; Fig. 4b; Supplementary Data 4). Garlic genes co-expressed with virus genes were significantly enriched in the GO functional terms associated with defense response, such as ‘defense response to insect’ (*P* = 8.90 × 10^−6^), ‘abscisic acid-activated signaling pathway’ (*P* = 6.13 × 10^−5^), and ‘regulation of defense response’ (*P* = 2.30 × 10^−12^) (Supplementary Figs 10 and 11). Many viruses depend on host heat stress proteins (HSPs) for folding, and host HSPs are frequently involved in viral infection [41]. Of the co-expressed genes, nine HSP genes showed co-expression with genes of garlic viruses B, D, and E and the shallot latent virus (*P* < 0.01), whereas two HSP genes were co-expressed with genes of garlic viruses A and X (Supplementary Figs 12 and 13), suggesting the potential involvement of these HSP genes in the viral infection of garlic. Further correlation analysis between the transcript abundance of co-expressed garlic and virus genes in clove cells identified three genes of which expression was correlated with those of garlic virus B and E genes, with a correlation coefficient of >0.7 (Fig. 4c and d; Supplementary Data 4); these genes encoded the disease-defense-related lectin protein [42]. Overall, co-expression analysis at the single-cell level identified numerous defense-responsive genes, providing an important basis for understanding the interactions between garlic and viruses.

**Figure 5:**
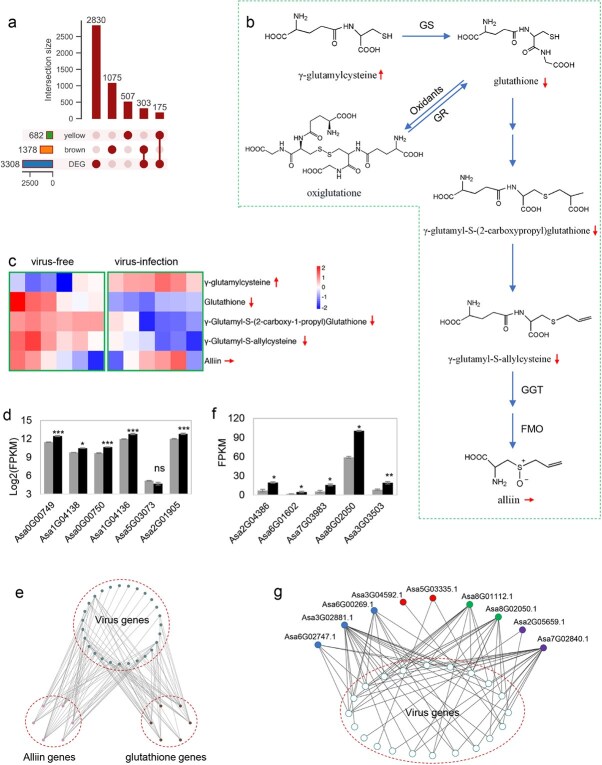
Interaction between the genes of garlic and viruses. (**a)** Upset Venn diagram showing the overlap number of co-expressed garlic genes in the yellow and brown modules and DEGs between virus-invasive and virus-free garlic. The horizontal histogram at the left shows the total gene number in the three investigated gene sets. The vertical histogram at the top shows the number of unique and common genes. The bright dots represent the presence of the genes in the gene sets listed on the left side, while the light dots represent their absence. The vertical column with only one bright dot represents unique genes in the corresponding gene set. The vertical column with more than one bright dot represents the common genes for multiple gene sets. (**b**) A putative pathway of biosynthesis and oxidant of glutathione, and its metabolism as the substrate of alliin. GS, glutathione synthetase; GR, glutathione reductase; GGT, gamma-glutamyltranspeptidase; FMO, flavin-containing monooxygenase. (**c**) Heatmap showing the difference in focused metabolites in six virus-free and six virus-invasive garlic individuals. Up, down, and horizontal arrows in the right of metabolite in (**b**) and (**c**) represent a significant increase, decrease, and no change for corresponding metabolite between virus-free and virus-infected garlic. (**d**) Expressed difference of five glutathione synthetase genes and one glutathione reductase gene between virus-free and virus-infected garlic. The *y* axis indicates the value of log_2_ (FPKM). For each gene, the left and right represent the log_2_ (FPKM) value of the gene in virus-free and virus-infected garlic, respectively. (**e**) A schematic diagram visualizes the co-expression between viral genes, alliin biosynthetic genes, and glutathione-related genes. (**f**) Expressed difference of five RNA silencing-related genes between virus-free and virus-infected garlic. ^***^, ^**^, and ^*^ in (**d**) and (**f**) indicate significance at 0.001, 0.01, and 0.05, respectively. (**g**) A schematic diagram visualizes the co-expression between viral genes and RNA silencing-related genes

We developed virus-free garlic plants using the method of tip shoot culture. Favorable bulb performances in virus-free plants highlighted the impact of viruses on swelling growth of garlic bulb (Supplementary Fig. 14). Further RNA sequencing and metabolomic analysis identified 156 differential metabolites and 3308 differentially expressed genes (DEGs) in virus-infected garlic clove, compared to that of virus-free garlic plants (Supplementary Data 5 and 6; Supplementary Figs 15 and 16). Of the co-expressed garlic genes in modules brown and yellow, 303 (22.0%) and 175 (25.7%) showed an expressed difference between cloves of virus-free and virus-invasive garlic (Fig. 5a), implying their involvement in the viral immunity response. Additionally, the metabolomic analysis indicated a significant downregulation in the glutathione level but a distinct increase in its precursor γ-glutamylcysteine in virus-infected garlic clove, with a content of 0.72- and 3.28-fold compared to that in virus-free garlic clove, respectively (Fig. 5b and c). Interestingly, there were five glutathione synthase and one glutathione reductase gene that co-expressed with virus genes, five of which displayed up-regulated expression in virus-invasive garlic clove (Fig. 5d and e), which seems to be contradictory with the reduction of glutathione content in virus-infected garlic clove. Alliin is biosynthesized from glutathione, and the reduction of glutathione caused a synchronous decrease in some intermediate metabolites for alliin biosynthesis (Fig. 5c). Notably, several genes in the alliin-biosynthetic pathway, (e.g. gamma-glutamyltranspeptidase genes and flavin-containing monooxygenase genes [43]), showed co-expression with virus genes in clove cells (Fig. 5e).

RNA silencing functions as a primary immune defense of viruses in plants, and some proteins including RNA-dependent RNA polymerases (RDRs), dsRNA-specific endoribonuclease Dicer or Dicer-like (DCL) proteins, argonaute (AGO) proteins, and aminophospholipid transporting ATPase 1 (ALA1) play pivotal roles in this immune process [44]. In this study, we identified five DEGs related with RNA-silencing between virus-free and virus-accumulated garlic plants (Fig. 5f), including one ALA1-like gene (*Asa2G04386.1*), two RDRs-encoding genes (*Asa3G03503.1* and *Asa8G02050.1*), one AGO-like gene (*Asa6G01602.1*), and one DCL-encoding gene (*Asa7G03983.1*). Besides, nine genes encoding the homologous proteins of AGO, ALA1, RDR, and DCL showed a co-expression with virus genes in clove cells (Fig. 5g). These findings implied a potential involvement of RNA silencing in viral immune defense of garlic.

## Discussion

Although the significance of cloves is great in garlic production because they are consumed as both edible and asexually reproduced organs, current understandings of clove growth remain limited. This study characterized the single-cell transcriptomic landscape of swelling cloves, and uncovered the cell differentiation trajectory of swelling clove. Recent single cell-RNA sequencing of garlic basal plate in which inflorescence begins formation has identified ten cell clusters, and they differentiate toward two cell branches [45]. Interestingly, cells at different branch show differential association with reproductive transition [45], indicating the divergency of function for these differentiated cells. Similarly, two cell developed paths that respectively start from C7 and C11 cells are identified in the swelling clove in this study. According to the cell trajectory, C1 cells are at the end of the differentiation road of clove cells. C1 cell-expressed-specifically genes display higher expression in the mature bulb-3 than in the other samples. Additionally, there are 58.9% of bulb-3 cells to be the C1 type (Supplementary Table S2). Because parenchyma cells are the most prevalent type in the flesh of mature clove, our evidences supported that C1 cells are parenchyma type. The single-cell transcriptomic atlas provides important resource for researching cell-specific gene expression of swelling clove, which is helpful for comprehensively elucidate the bulb organogenesis.

A significant advancement of this study was the discovery of the invasive virus activity features in swelling cloves at single-cell resolution. Viruses are widespread in plants, and viral diseases have profound effects on crop production. Plant stem cells remain virus-free due to the protective *WUSCHEL*-mediated antiviral immunity system [24]; however, cells become susceptible to viral intrusion as they differentiate and develop. The exact nature of virus activity in cells during this process has remained largely unknown. Cultivated garlic is completely sterile, and once a garlic plant is infected by viruses and adapts to their infection, these viruses persist in its clonal offspring indefinitely. Therefore, garlic serves as an ideal species for studying the evolutionary adaptation of plants to virus infection. scRNA-seq effectively detects virus genes even though they typically lack polyA sequences [46], suggesting that scRNA-seq is a valuable tool for analyzing the interaction between pathogenic microorganisms and host organisms at the single-cell level. Herein, scRNA-seq analysis revealed the active characteristics of invasive viruses in cloves under swelling growth at single-cell resolution. Limited viruses appeared in the cells of young cloves, suggesting that, of reads sequenced from cloves, those aligned into viral genes were from the viruses, but not from viral elements of garlic genome. Furthermore, the method of culturing the young cloves *in vitro* has been developed as a new and straightforward strategy for removing viruses from garlic, which could potentially replace the labor-intensive isolation and time-consuming culturing of shoot tips.

Virus infection often involves hijacking host genes to complete their replication, leading to an expression response to infection. Traditionally, a combination of yeast two-hybrid screening, co-IP, and/or mass spectrometry has been used to identify the interaction partners of viral proteins [47]. Recently, a TurboID-based proximity labeling approach has been proposed for detecting host factors involved in plant virus replication [41]. In the present study, many garlic genes have been identified with a co-expression with virus genes at the single cell level, and 23.2% of them were further compared by an expression comparison between virus-free and virus-infected garlic. In our transcriptomic comparison, because of the usage of virus-invasive garlic that experiences a perennial accumulation of viruses, there were some stress-responsive genes that might have adapted to the stress condition of virus infection or some genes that show a transient response to virus invasion; accordingly, it is difficult to detect their responses to virus invasion in our transcriptomic comparison. Additionally, transcriptomic analysis quantifies the gene expression level by estimating the average transcript abundance of gene in all cells of investigated sample, and its sensitivity in identifying DEGs is poorer than the single cell investigation. Notably, the virus-infected garlic used for transcriptome analysis in this study accumulates numerous viruses, which probably possess an antagonistic effect in inducing the expression of some garlic genes. All these are potential reasons for the fact that the expression response of >70% of co-expressed genes to viruses fails to be detected in the transcriptomic comparison between virus-free and virus-accumulated garlic.

Even so, some virus infection-responsive glutathione synthase/reductase genes are identified in this study. In plants, glutathione is deemed a master antioxidant in the regulation of resistant and susceptible host–plant virus interaction [23], because it can enable precise direct or indirect control of reactive oxygen species, which often accumulate or produce at high levels during biotic stress, thereby reducing damage to the cells [48, 49]. In the present study, the co-expression of glutathione-related genes with virus genes and their up-regulated expression in virus-invasive garlic suggest a response of glutathione-related genes to virus invasion in garlic. Theoretically, an increase of 3.28-fold in precursor and a higher expression level of enzyme genes for catalyzing the precursor to generate glutathione should increase glutathione level accumulated in virus-invasive garlic. However, the actual content of glutathione is lower in virus-infected garlic than in virus-free garlic, and a mechanism underlying this unexpected finding need to be further explored. Furthermore, this study identifies nine genes encoding the homologous proteins involved in RNA-silencing. These findings imply a complex defense mechanism against the virus invasion in garlic. Altogether, this study proves co-expression analysis between the genes of viruses and host plants at the single cell level to be an alternative approach for identifying virus infection-responsive genes.

Notably, this study focuses on the overall landscape of accumulated viruses in garlic. Significant impact on gene expression in virus-accumulated garlic has been found by comparing to virus-free garlic in which all viruses are removed. However, it is difficult to determine which virus is crucial in influencing the bulb growth, although several viruses have been identified to be prevalent in bulb. This study reveals a negative effect of accumulated viruses to bulb growth, which is accordant with the finding of previous study [20]. Additionally, the accumulation of various viruses leads to a significant decrease in the precursors of alliin, indicating a potential impact of them on garlic quality. Because viruses display few activities in meristem cells, and are active in cells of clove under medium and late-term of development, the impact of viruses on bulb yield and quality may take place mainly in the medium and late-term of clove swelling, which provides a guidance for the garlic cultivation in resisting viral disease.

## Materials and Methods

### Plant materials and phenotype

To estimate the accumulation of viruses in garlic variety Ershuizao, we performed an RNA sequencing for its clove. After filtering the reads that could mapped into garlic genome, 19.7% of reads were found from known viruses by searching against the NR database (Supplementary Fig. 17), using the software diamond [50], with default parameters. Therefore, the cloves of this material were deemed to be qualified for conducting scRNA-seq. From 1 January 2022, three garlic individuals were monitored daily for clove development until the collection of all three samples. Subsequently, the cloves, referred to as bulb-1, bulb-2, and bulb-3, were collected on 25 January, 10 March, and 29 March, respectively, from 10, 3, and 3 individuals, respectively.

### Sample preparation and protoplast isolation

To isolate protoplasts, all cloves from 10 individuals (bulb-1), 3 individuals (bulb-2), and 3 individuals (bulb-3) were mixed and utilized as the sample for the corresponding growth stage. For protoplast isolation, freshly washed samples were initially cut into small pieces with a sharp blade and promptly placed in an enzymatic digestion solution comprised of 3% (w/v) cellulase R-10, 1.5% crude granzyme R-10, 0.3% pectinase Y-23, 0.25% BSA, 5 mM MES, and 8% (w/v) mannitol (free of Ca^2+^ and Mg^2+^). Care was taken to ensure that the tissue was completely submerged. Petri dishes containing the sample and digestion solution were sealed with film and placed on a constant-temperature metal bath at 25°C for approximately 30 min for complete tissue submersion. Subsequently, enzymatic digestion was performed at 25°C and 200 rpm for 1–2 h. The tissue was then filtered through a 40-μm cell sieve, washed with WI solution, and filtered again until the volume reached 10 ml. The filtrate was then divided into two tubes and centrifuged at 100 × g for 7 min at room temperature. The pellet was resuspended in WI solution, examined under a microscope, and counted. Following this, the suspension underwent centrifugation at 200 × g for 7 min at 25°C. This process was repeated after washing with WI solution. Subsequently, the protoplasts were rinsed 3–4 times with 8% mannitol at 25°C.

### scRNA-seq library construction and sequencing

The library was constructed utilizing a Chromium Next GEM Single Cell 3′ GEM, Library, and Gel Bead Kit v3.1 (10x Genomics, USA) following the manufacturer's instructions. To ensure single-cell resolution, cells were delivered at a limited dilution to produce single-cell gel beads-in-emulsion (GEMs). The 10× Genomics instruments mix individual cells with individual GEMs via the oil phase to form small water-in-oil droplets, which then disrupt the cell membrane and release the mRNA from the cells. The free mRNA mixes with the water in the droplet and comes into contact with reverse transcriptase, nucleic acid primers bound to gel beads, and dNTP substrate. A reverse transcription reaction then occurs in which the mRNA binds to the labeled DNA molecules on the gel beads and reverse transcribes the cDNA in the presence of reverse transcriptase. The full-length cDNA is fragmented by enzymatic cleavage, and the read 1 primer is added to the molecule during GEM incubation, followed by end-repair, A-tail, and adapter ligation of the read 2 primer sequence. Immediately thereafter, library construction was completed by PCR reaction incorporation of Index, P5, and P7. Finally, these libraries were subjected to paired-end sequencing using the Illumina NovaSeq 6000 system (Illumina, San Diego, USA).

### Data analysis and clustering

The raw data from scRNA-seq were processed using the official 10x Genomics software CellRanger (v7.0.1) (https://support.10xgenomics.com/single-cell-gene- expression/software/pipelines/latest/what-is-cell-ranger) for quality statistics and comparison to the reference garlic genome (GenBank accession No.: GCA_014155895.2). Additionally, the viral genome was added to the references to quantify the expression levels of virus genes. Briefly, scRNA-seq reads were aligned into the NR database of the National Center for Biotechnology Information (NCBI), and the number of reads from each virus was counted. The top six virus genomes, i.e. garlic virus A (GCA_000848665.1), garlic virus B (GCA_000928855.1), garlic virus D (GCA_000915015.1), garlic virus E (GCA_000852345.1), garlic virus X (GCA_000856365.1), and the shallot latent virus (GCA_031105625.1), were then added as references for analysis using CellRanger software (v7.0.1). The software allowed quantification of high-throughput single-cell transcriptome data and further quality control of the data through the Seurat (v4.0.0) software package [30]. To eliminate low-quality cells and potential multiplets (a major issue in droplet-based experiments), several criteria were employed to further control the data quality: (i) Only cells expressing a gene count between 500 and 10 000 were considered; (ii) Cells with unique molecular identifiers (UMIs) greater than 50 000 and less than 500 were filtered out; (iii) Genes expressed in fewer than three cells were removed. Furthermore, the DoubletFinder software package (version 2.0.2) [51] was utilized to identify potential doublets. Following the application of these quality control standards, 19 681 single cells were included in downstream analysis. The library size was normalized using the NormalizeData function in Seurat to obtain normalized counts. Using the FindVariableGenes function within the Seurat software package, highly variable genes (HVGs) were selected. Principal component analysis was performed on the expression profiles of the HVGs. Subsequently, the batch effect was removed using the mutual nearest neighbor approach from the Batchelor software package [52]. Then, utilizing the FindClusters function within the Seurat software package, cells were clustered based on their gene expression profiles. The results were visualized on a two-dimensional space using the two-dimensional uniform manifold approximation and projection (UMAP) algorithm.

### Identification of expressed genes in various cell clusters

For each gene, the number of cells in which the gene was expressed was determined and used to calculate the ratio of gene-expressed cells in one cell cluster (pct1) and the second cluster (pct2). Then, the genes expressed in each cell population were then identified using the Seurat Findallmarker function (test.use = Wilcox, logfc.threshold = 0, min.pct = 0.25). Additionally, we selected 100 genes with the highest expressed differences as marker genes to infer the cell type of each cluster. Finally, the genes were visualized using the VlnPlot and FeaturePlot functions.

### Predicting cell type by orthologous analysis and functional enrichment analysis

Genes whose expression was identified in meristematic cells by the FISH assay were collected, and their orthologs of garlic were identified and used to infer the cell type using AddModuleScore [53]. Briefly, the AddModuleScore function from the Seurat package [30] was used to calculate the expression score of a gene set in a single cell, which, combined with randomized comparisons of control features, provided a quantitative assessment of the gene set at the cellular level. Additionally, enrichment analysis of cell-expressed genes was performed. Specifically**,** the R package ClusterProfiler was used to assign GO functions to all cell-expressed genes [54]. GO annotations were available from the NCBI (http://www.ncbi.nlm.nih.gov/) and GO (http://www.geneontology.org/) databases. Common features of genes in gene collections in terms of biological processes, molecular functions, and cellular composition were obtained. The name for the cell type was determined by referencing ‘Esau's Plant Anatomy’ [55].

### Pseudo-time analyses

We utilized the Monocle2 package (v2.9.0) to simulate the dynamics of pseudo-time development based on the expression patterns of marker genes [56]. Raw counts were first converted from Seurat objects to CellDataSet objects using the import oding sequence (CDS) function in Monocle. The differalGeneTest function of the Monocle2 package was employed to select sorting genes (q-val < 0.01) that may provide information for sorting cells along pseudo-time trajectories. The reduceDimension function was utilized to conduct a reduced-dimensional clustering analysis, followed by trajectory inference using the orderCells function with default parameters. Gene expression was plotted visualized using the plot_genes_in_pseudotime function to track changes in pseudo-time. Branching points were chosen for analyzing branches of the differentiation trajectory, and the pseudo-time-dependent or branching-dependent genes were identified using the Branched Expression Analysis Modeling method (BEAM) [57]. Genes with significant branching dependence were visualized using the ‘plot_genes_branched_heatmap’ function of BEAM.

### RNA velocity and CytoTRACE analysis.

To perform RNA velocity analysis, the Python script velocyto.py (https://github.com/velocyto-team/velocyto.py) was applied to the Cell Ranger output folder to recalculate spliced reads and unspliced reads [58]. The likelihood-based dynamical model and velocity graph were constructed using scVelo (https://scvelo.readthedocs.io/) to calculate RNA velocities (transcription, splicing, and degradation rates) for single cells [59]. The velocity fields were projected onto the UMAP embedding layer found by Seurat. CytoTRACE (version 0.3.3) [60] was utilized to predict the differentiation states of cells from scRNA-seq data, with the parameter: enableFast = TRUE. The result from CytoTRACE analysis was visualized using the plotCytoTRACE function of the program.

### Producing virus-free garlic

We generated the virus-free garlic by the shoot-tip culture methods using approximately three year-time. Briefly, shoot tips were isolated from the aseptic cloves of ‘Chalingzipisuan’, a landrace with considerable accumulation in the viruses Nov. 2020, and cultured in culture medium at 25°C and under a 16 h/d photoperiod. In Oct. 2021, garlic seedlings were grown in an insect-free greenhouse, and real-time quantitative PCR was used to estimate their viral content. The virus-free garlic plants were retained and expanding propagation in the experimental farm covered by fly net in October 2022.

This study also developed a new method for producing virus-free garlic by the *in vitro* culture of young clove. Briefly, young cloves from cv. Ershuizao were collected in March 2023 and dipped in sterile water with 20 min. Then, these cloves were transferred into the 20% sodium hypochlorite solution. After soaking 15 min, the cloves were washed thrice using sterile water. Subsequently, these cloves were transferred onto the MS medium, and cultured at 25°C and under a 12 h/d photoperiod. After taking roots, the cultured seedlings were transplanted in an insect-free greenhouse.

### RNA-sequencing

Virus-free garlic and its parent accumulated viruses were grown in fly net-covered farm, and their swelling bulbs were collected for RNA sequencing. Total RNAs extracted from each replication were individually used to construct cDNA libraries using a NEBNext UltraTM RNA Library Prep Kit for Illumina (New England BioLabs, Ipswich, MA, USA) according to manufacturer instructions. Subsequently, the cDNA library was then subjected to paired-end sequencing on the Illumina sequencing platform using a HiSeq PE Cluster Kit v4 cBot (Illumina, San Diego, CA, USA). After removing the adapters and low-quality base sequences using fastp software, the high-quality reads were aligned with the garlic genome (GenBank accession: GCA_014155895.2) using hisat2 software (version: 2.2.1.0) [61], with default parameters. Gene expression was calculated using FPKM (Fragments per kb per Million reads) values [60]. The DESeq2 package in the R software [62] was used to identify DEGs between virus-free and virus-accumulating garlic bulbs, and defined genes with a Log2FC (Log2 fold change) > 1 as significant DEGs (*P* < 0.05).

### LC–MS-based untargeted metabolomic analysis

Six virus-free and six virus-accumulated garlics were planted with the same method used for RNA sequencing, and their bulbs were individually used for metabolomic analysis as six replications. Approximately, 60 mg of tissue for each sample was used to extract the metabolites. The samples were extracted by ultrasonic extraction with 90% ethanol, centrifuged to remove the supernatant, and reconstituted with water after nitrogen blowing. After derivatization with dansulfonyl chloride, the samples were separated on a Waters HSS T3 column (100 × 2.1 mm, 1.8 μm), and the temperature of the column was maintained at 45°C. The LC–MS metabolic profiles were collected by mass spectrometry using an electrospray ionization source in both positive and negative modes. The analysis was conducted using the following parameters: spray voltage set to 3800 V, capillary temperature at 320°C, auxiliary gas heater temperature maintained at 350°C, sheath gas flow rate at 35 Arb, auxiliary gas flow rate at 8 Arb, S-lens RF level set to 50, mass range defined within m/z, full MS resolution at 70 000, MS/MS resolution at 17 500, and NCE/stepped NCE set to 10, 20, and 40. Subsequently, accurate identification of metabolites was achieved based on comprehensive analysis of RT and m/z values as well as MS/MS profiles. The data matrix was also exported as an Excel file for subsequent analysis.

### Phylogenetic analysis of a candidate protein

Multiple sequence comparisons were first performed using the Clustal program [63]; then an unrooted phylogenetic tree was constructed using the neighbor-joining method and bootstrap testing with 1000 replications was performed using the MEGA software [64].

### Co-expressed analysis

Co-expression between garlic and virus genes was analyzed using weighted gene co-expression network analysis [65] in the R software package. Genes with co-expression networks in all clove cells were constructed and gene modules associated with specific traits were identified according to previous descriptions [66]. Then, co-expressed transcripts were clustered into modules using the following parameters: a minimum module size of 30 genes, and merged of modules if they shared >25% similarity. The correlation between garlic and virus genes in clove cells was estimated using Pearson correlation.

### RNA FISH assay

Freshly collected clove tissues were fixed in a formaldehyde-acetic acid-ethanol fixative solution (50% ethanol, 5% acetic acid, and 3.7% formaldehyde) at 4°C overnight, dehydrated with ethanol from 50% to 100%, and embedded in wax. Tissue paraffin blocks were then sliced by a slicer with a thickness of 4 μm, fished by a spreading machine, and baked in an oven at 62°C for 2 h. After dewaxing, dehydration, and repair treatment, the section was digested using protease K (20 ug/ml) at 37°C. Then, the endogenous peroxidase in the section was removed by adding 3% methanol-H_2_O_2_, incubating at room temperature for 15 minutes. Antisense sequences of *Asa4G03456.1* (5’-CUGUGCAGACUCUUCAAUAACAU-3′) and *Asa7G05865.1* (5′-CUUGAAAUCCCAACAUCGGGAUGCACUUGC-3′) were synthesized using the solid phase phosphoramidite triester method to produce RNA probes, which were labeled by digoxigenin. The sense sequences of *Asa4G03456.1* (5′- AUGUUAUUGAAGAGUCUGCACAG-3′) and *Asa7G05685.1* (5′-GCAAGUGCAUCCCGAUGUUGGGAUUUCAAG-3′) were synthesized and labeled by digoxigenin and then used as the control probes. RNA *in situ* hybridization for *Asa7G05865.1* and *Asa0G05866.1* using their antisense and sense probes was performed in the samples of bulb-1. Briefly, after prehybridization at 37°C for one hour, the prepared section was performed for hybridization by adding the 6 ng/μl probe solution overnight at 37°C. Next, after washing the hybridization solution, the section underwent a serum blocking treatment using normal rabbit serum at room temperature for 30 min, and then an anti-Digoxin antibody (anti-DIG-HRP) labeled by horseradish peroxidase was added. Incubating at 37°C for 50 min. Subsequently, after performing a treatment of tyramide signal amplification, the section was incubated in the solution of 4′,6-diamidino-2-phenylindole (Dapi) under dark conditions for 8 min. Finally, the transfected section of the prepared clove was observed using the Leica Nikon Eclipse CI spectral confocal microscope under the channel 488, and the blue and green fluorescence signals represented the observations for the nucleus and target gene-expressed RNA in the cell, respectively.

### Real-time fluorescent quantitative PCR

Leaf was collected from the garlic seedlings cultured by young cloves *in vitro*, and the garlic seedling germinated from a normal clove was used as a control. Additionally, young cloves were collected every seven days (cv. Ershuizao), from 19 February to 25 March, when the clove was before swelling and under swelling, respectively (shown in Fig. 1g). Each sample was performed in six replicates, and all tissues were used for RNA extraction individually. And reverse transcription was performed using RNA as a template, followed by qRT-PCR using the 18S ribosomal RNA gene as an internal control (see Supplementary Table 5 for primer sequences). For each sample, all six individuals were used as six biological replications, and the relative expression levels were estimated according to the method described by Livak and Schmittgen [67].

## Supplementary Material

Web_Material_uhae365

## Data Availability

The raw scRNA-seq data reported in this paper have been deposited in the Genome Sequence Archive [68] in the National Genomics Data Center, China National Center for Bioinformation/Beijing Institute of Genomics, Chinese Academy of Sciences (GSA: CRA011872). The sequence reads of transcriptome and the expression data have been deposited in the NCBI GEO database under the accession number GSE185962.
