# Supplementary material for: A single-cell transcriptomic atlas reveals the cell differentiation trajectory and the response to virus invasion in swelling clove of garlic
Source: Hortic Res. 2025 Jan 3;12(4):uhae365. doi: 10.1093/hr/uhae365 (PMC11894531; doi:10.1093/hr/uhae365)
Supplement: Web_Material_uhae365 [file web_material_uhae365.zip › Supplementary Figure.docx]

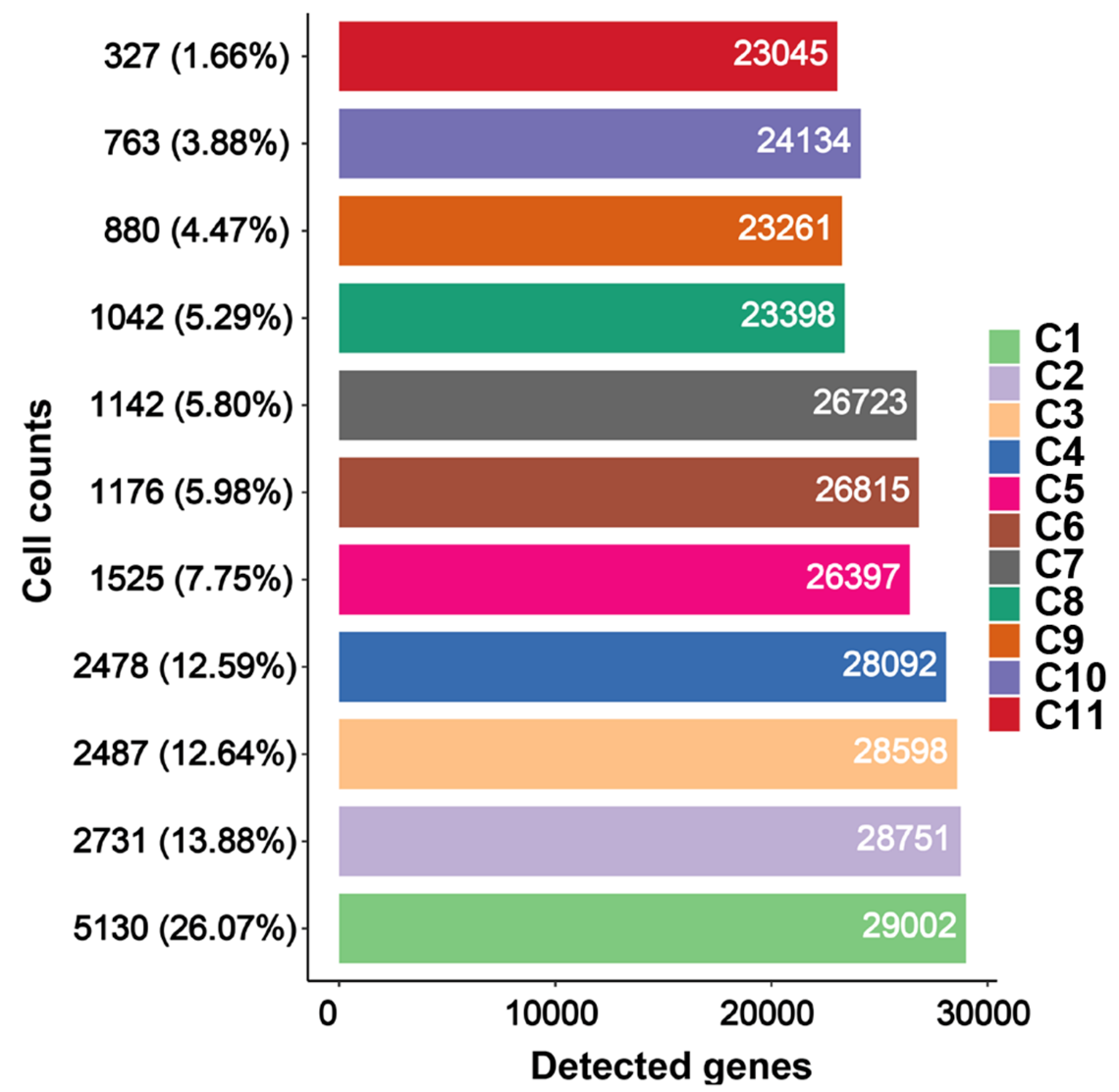


**Fig. S1:** Cell number, proportion, and expressed genes detected in each cell cluster from clove.


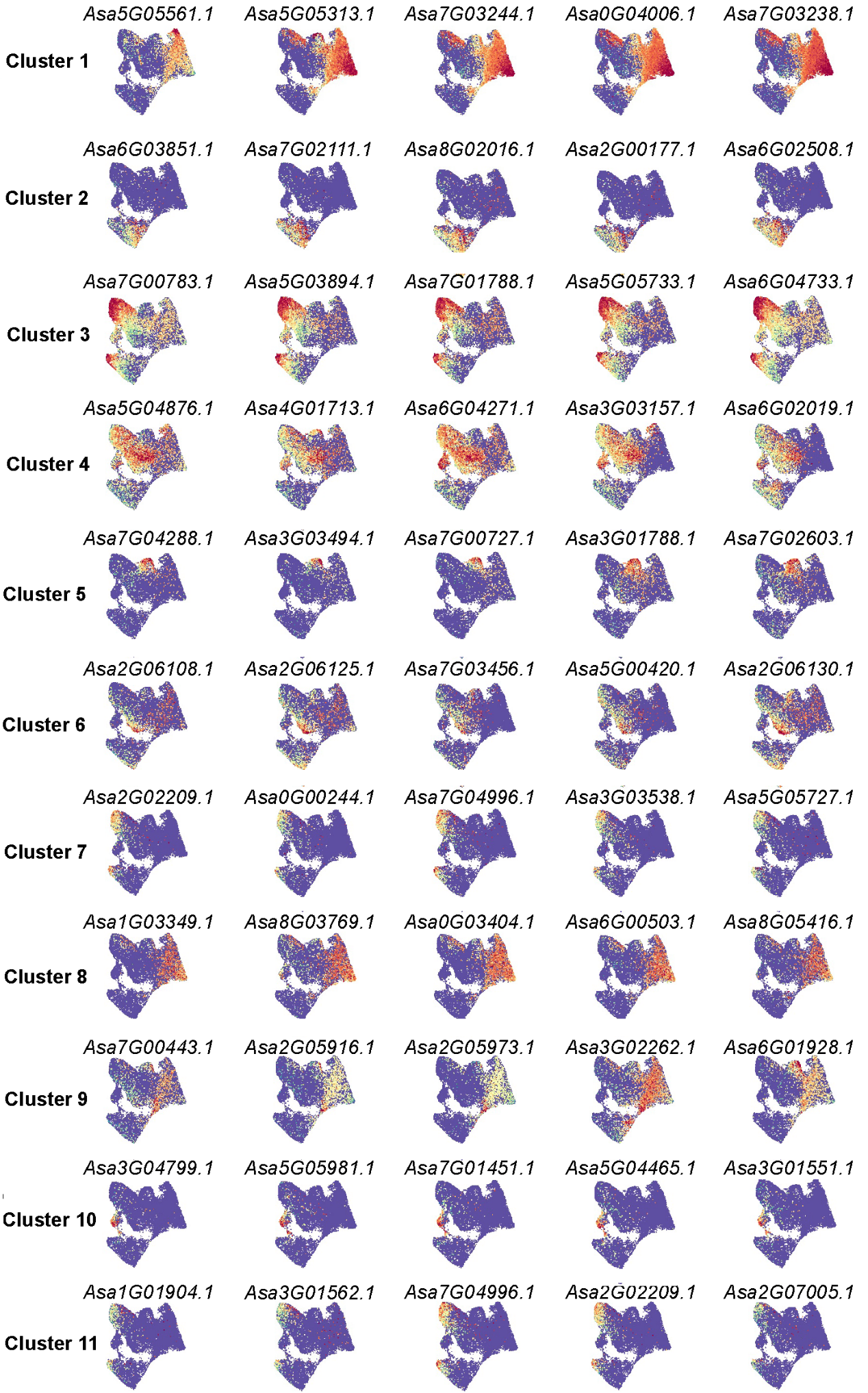


**Fig S2:** Distribution feature of representative genes of each cluster on UMAP plot.


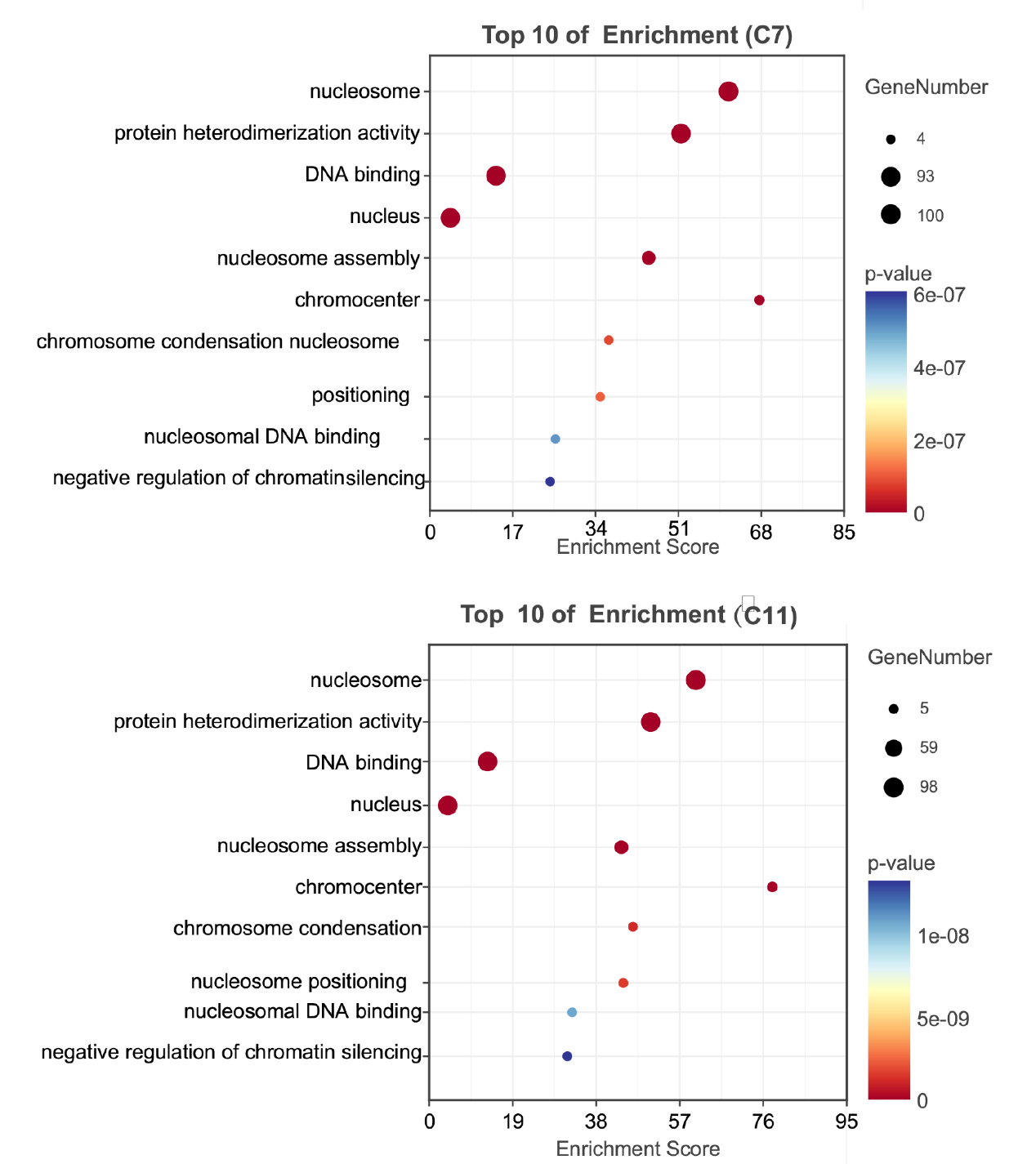


**Fig. S3:** Enrichment of GO terms by the top 100 highly expressed genes in cells of C7 and C11.


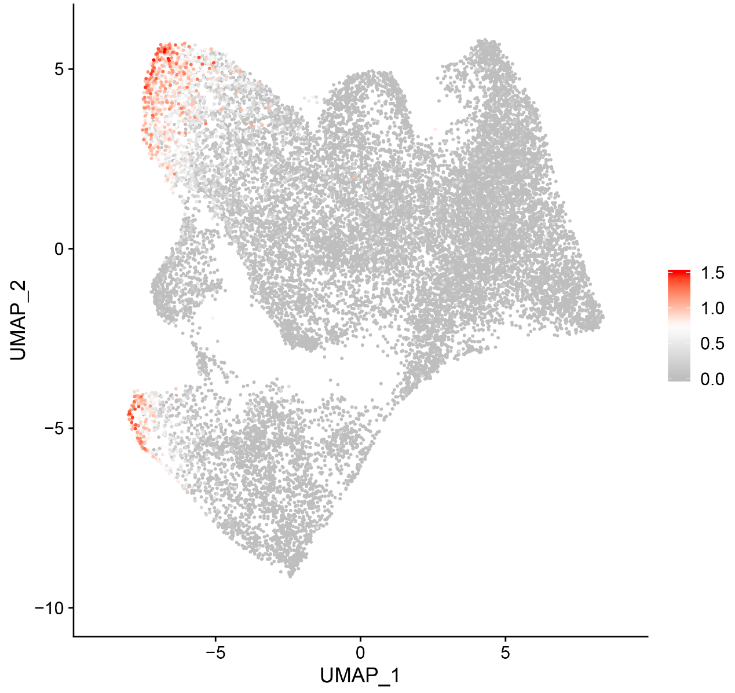


**Fig S4:** UMAP plots from the AddModuleScore analysis using garlic genes whose orthologs were expressed specifically in meristermatic cells.


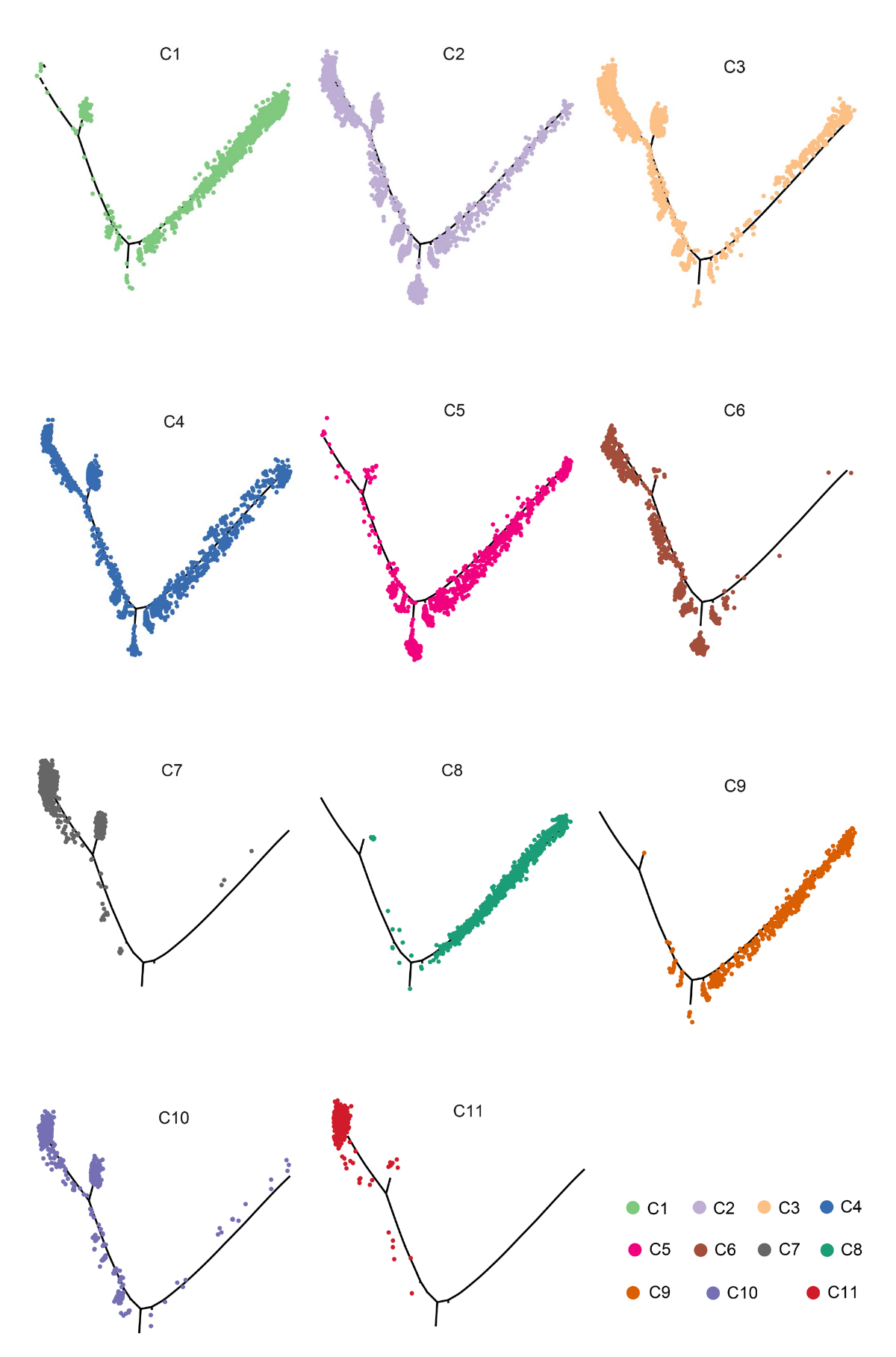


**Fig S5:** Pseudo-time trajectory of cells of each cluster in developmental cloves. Each dot represents a single cell.


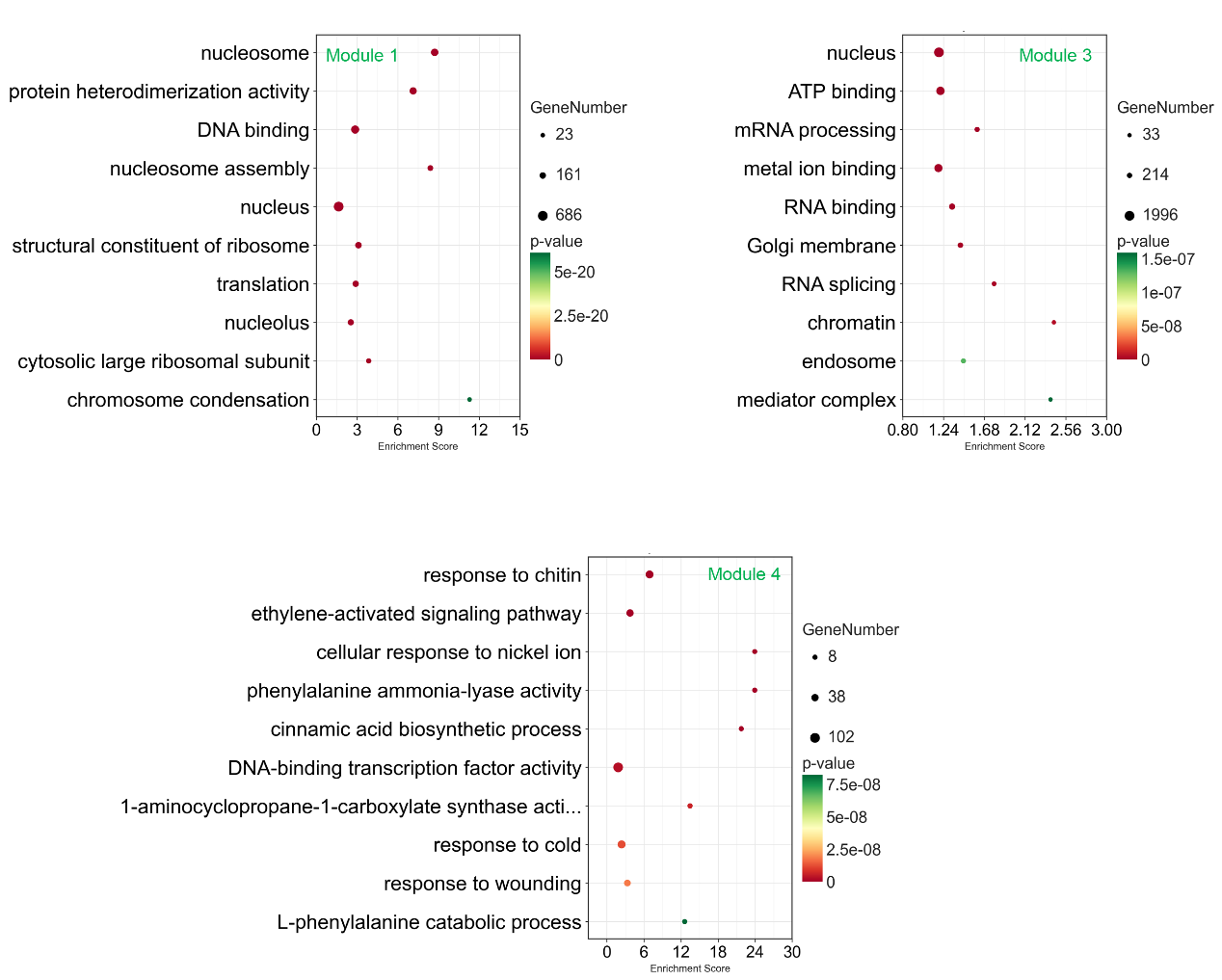


**Fig S6:** Enrichment of GO terms by genes with three different expressed module in differentiated cells compared to in cells at pre-branch.


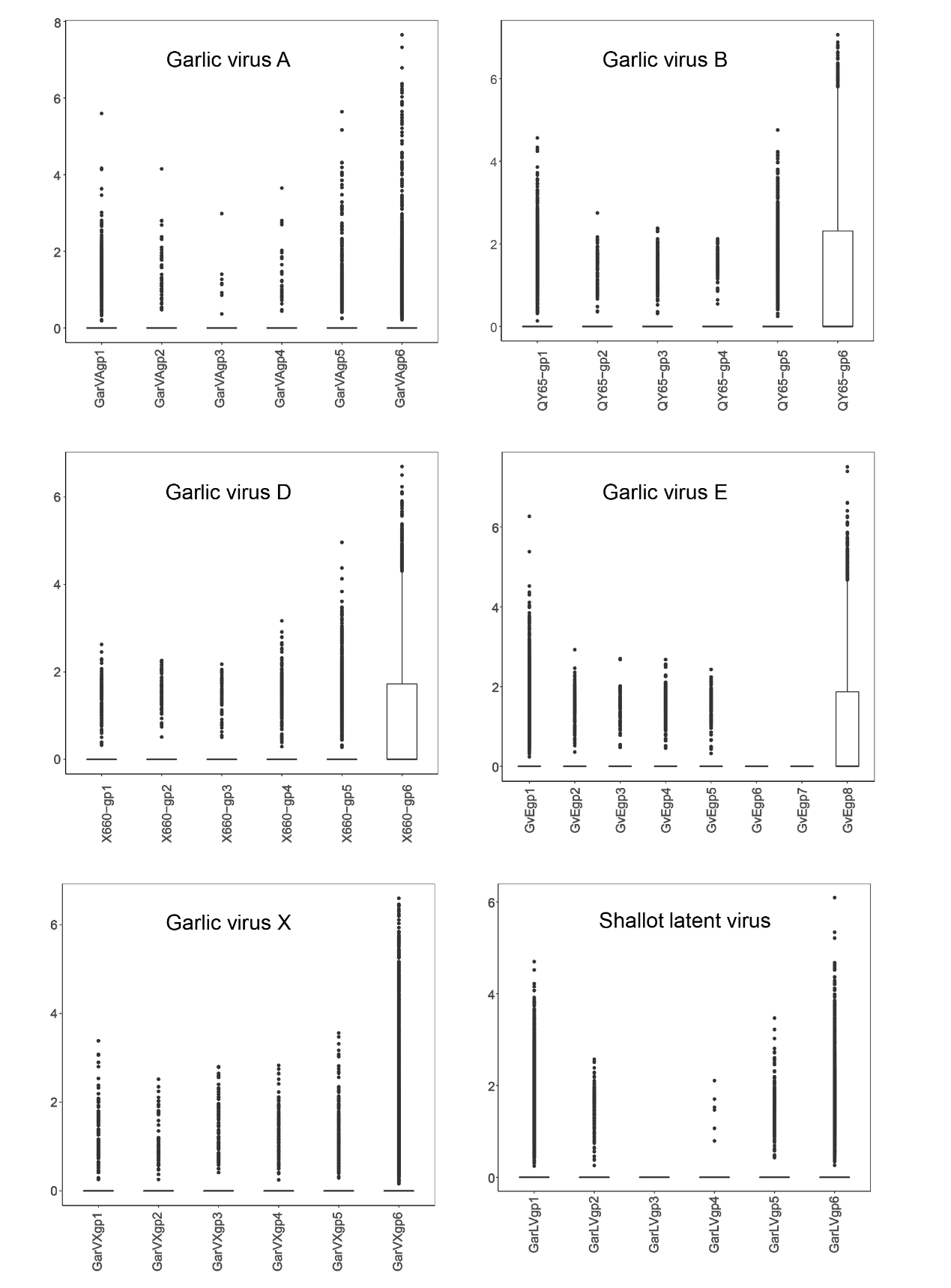


**Fig S7:** Boxplot showing the expression level of all genes from six main viruses in 19,681 cells of cloves


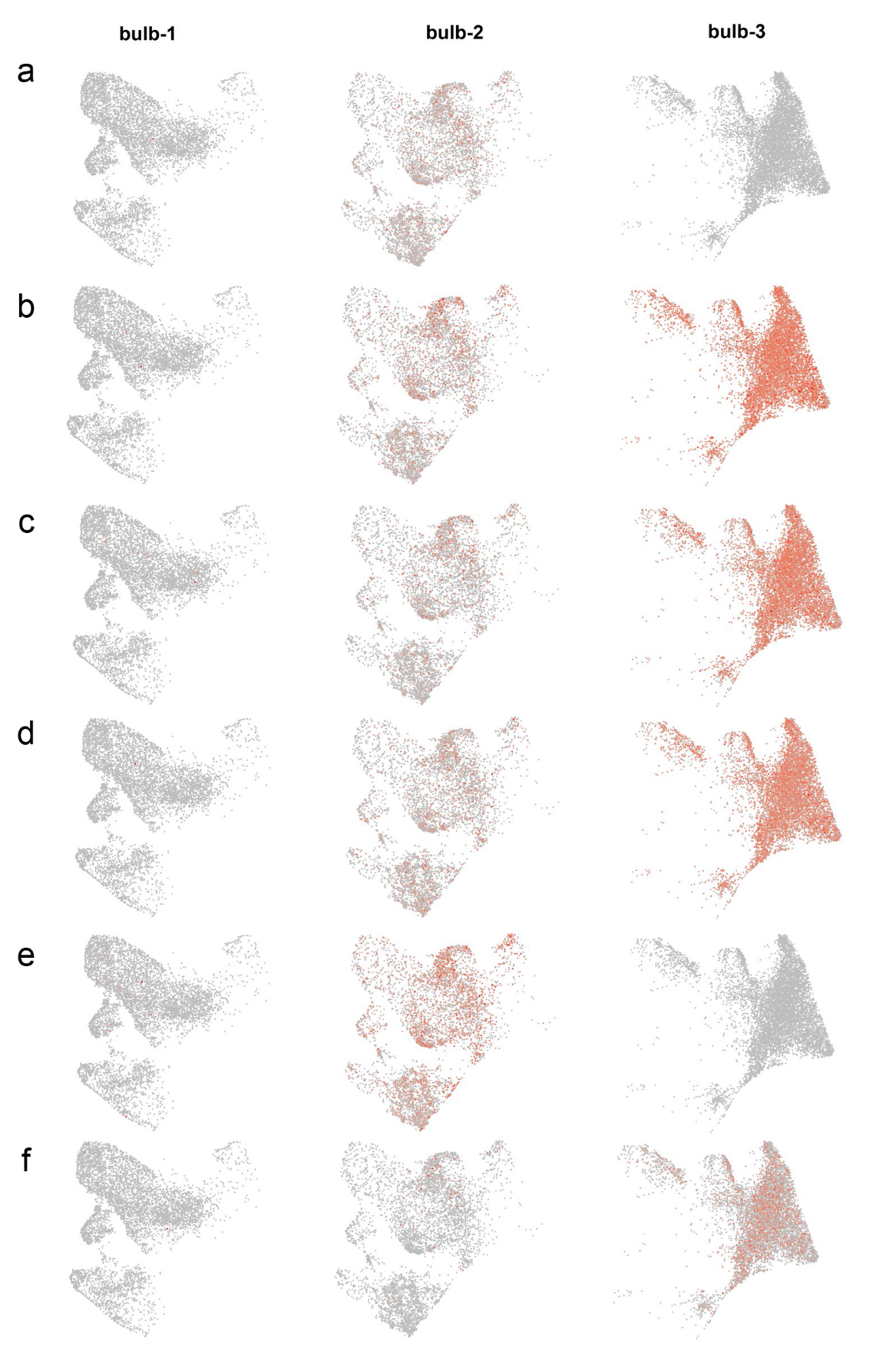


**Fig. S8:** UMAP of genes from six viruses in clove cells of investigated bulb-1, bulb-2, and bulb-3. *GarVAgp6* (**a**), *QY65.gp6* (**b**), *X660.gp6* (**c**), *GvEgp8* (**d**), *GarVXgp6* (**e**), and *GarLVgp6* (**f**) are used to estimate the activity of Garlic virus A, B, D, E, X, and Shallot latent virus, respectively., respectively.


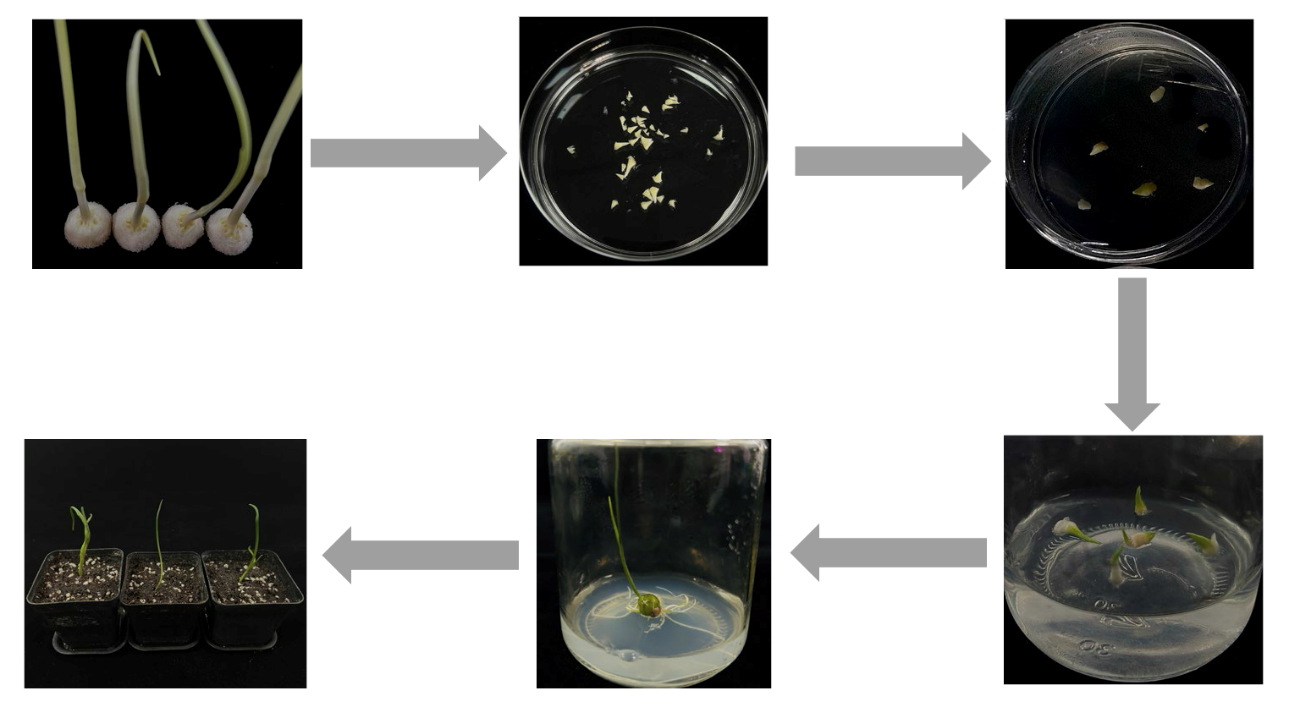


**Fig. S9:** A flow for producing virus-free garlic seedlings by culture *in-vitro* of young clove


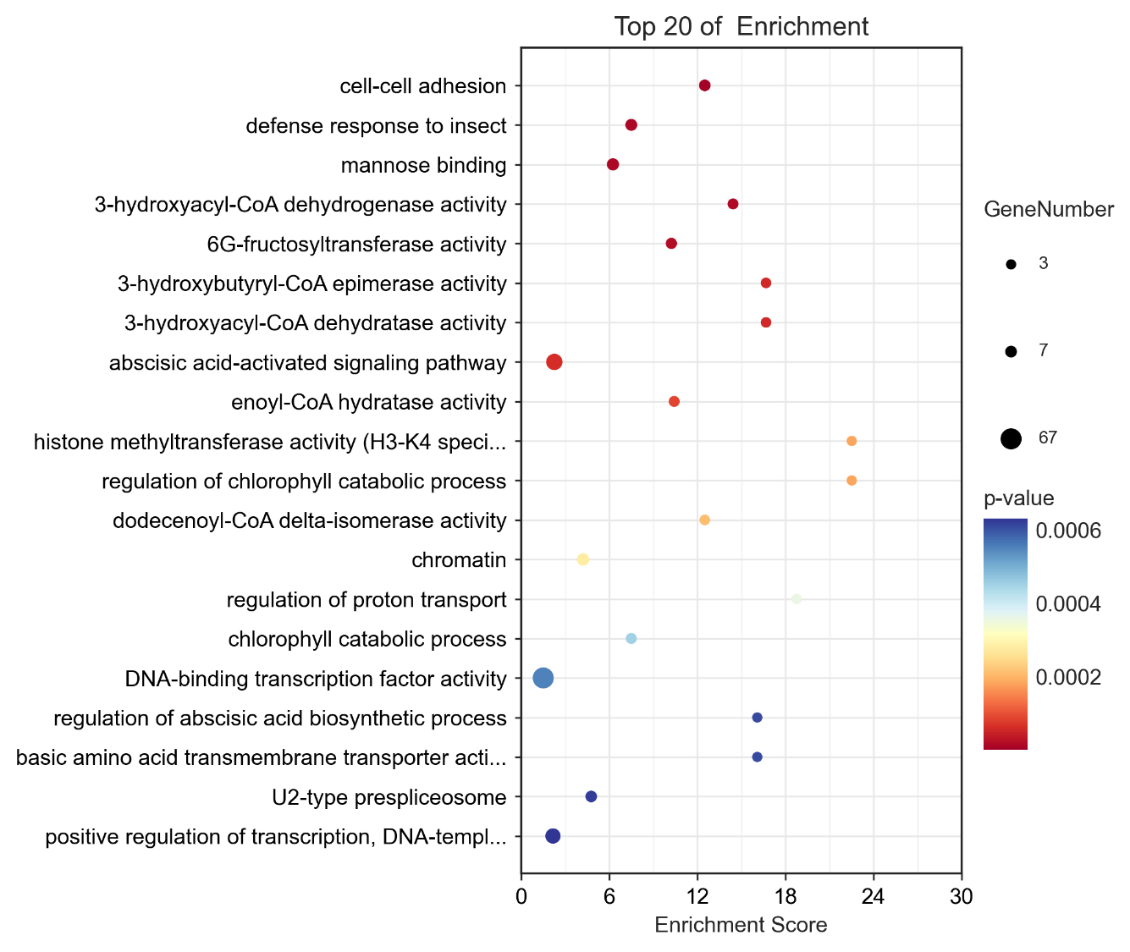


**Fig. S10:** Enrichment of GO terms by 1378 garlic genes co-expressed with genes of Garlic viruses B, D, E, and Shallot latent virus.

**
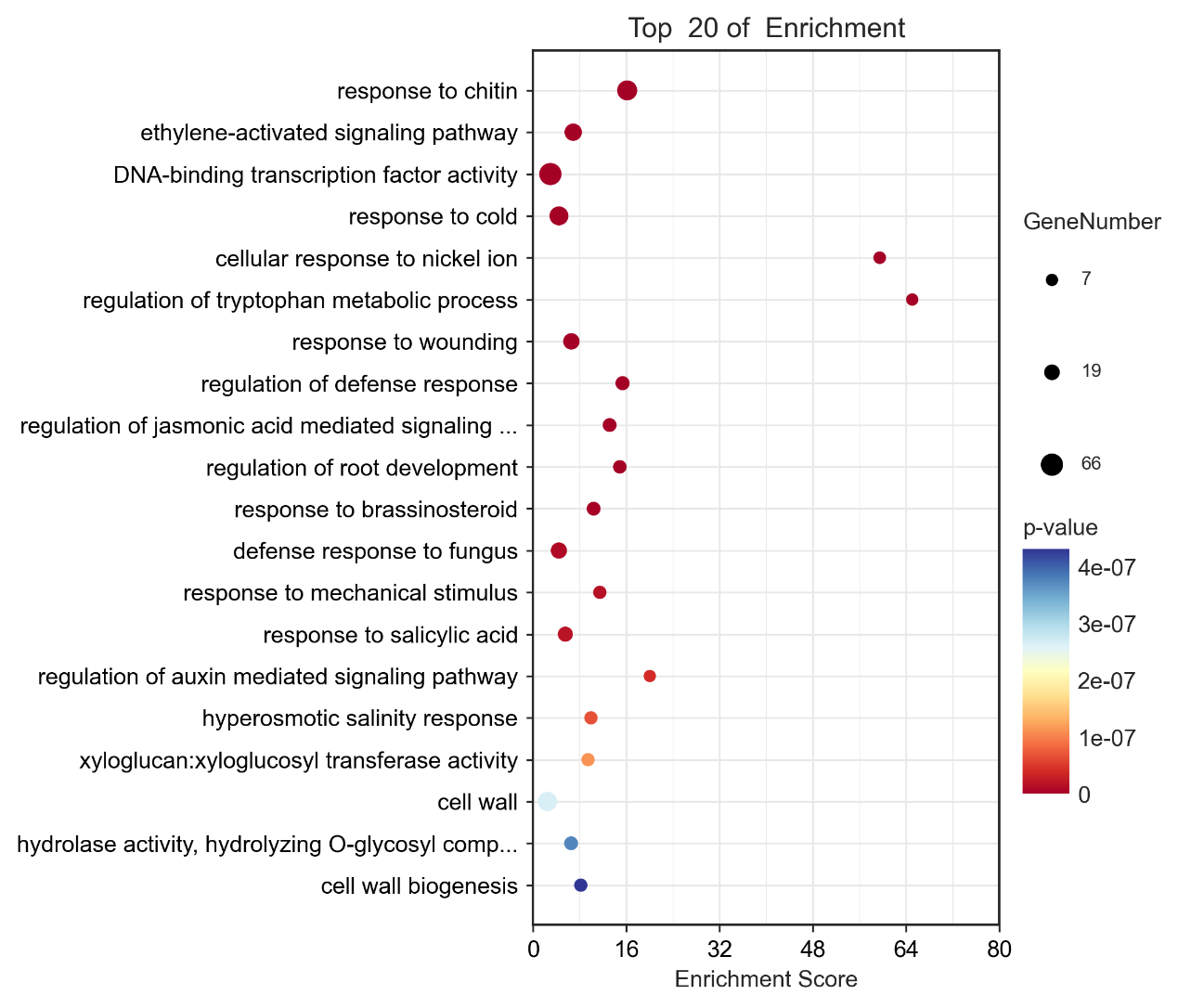
**

**Fig. S11:** Enrichment of GO terms by 1378 garlic genes co-expressed with genes of Garlic viruses A and X.

**
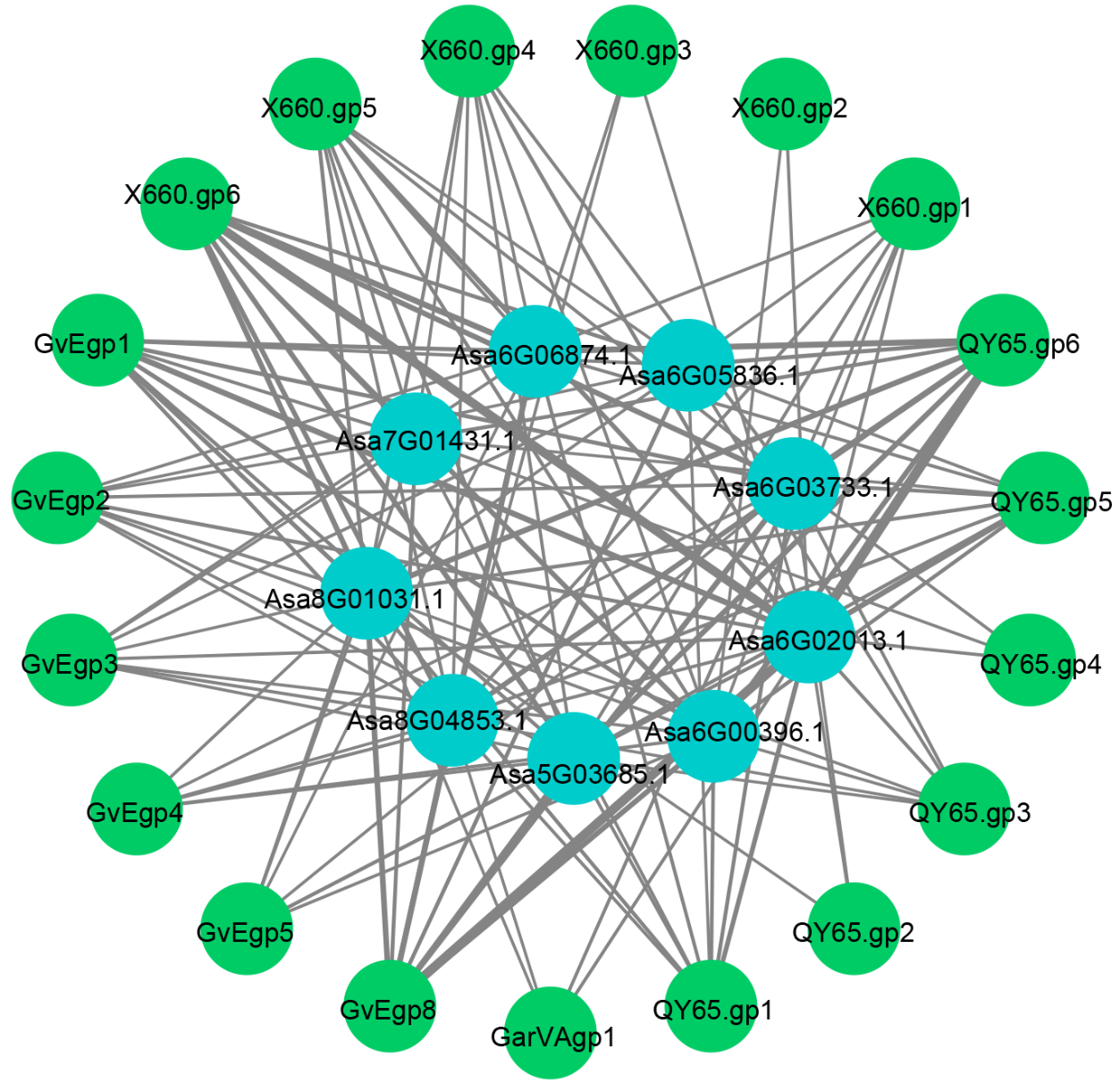
**

**Fig. S12:** Co-expression network of garlic heat stress protein-encoding genes and virus genes. *GvEgp1,* *GvEgp2*, *GvEgp3*, *GvEgp4*, *GvEgp5*, and *GvEgp8* are genes of Garlic virus E; *GarVAgp1* is gene of Garlic virus A; *QY65.gp1*, *QY65.gp2*, *QY65.gp3*, *QY65.gp4*, *QY65.gp5*, and *QY65.gp6* are genes of Garlic virus B; *X660.gp1*, *X660.gp2*, *X660.gp3*, *X660.gp4*, *X660.gp5*, and *X660.gp6* are Garlic virus D. The thickness of line between two genes indicates the co-expression level.


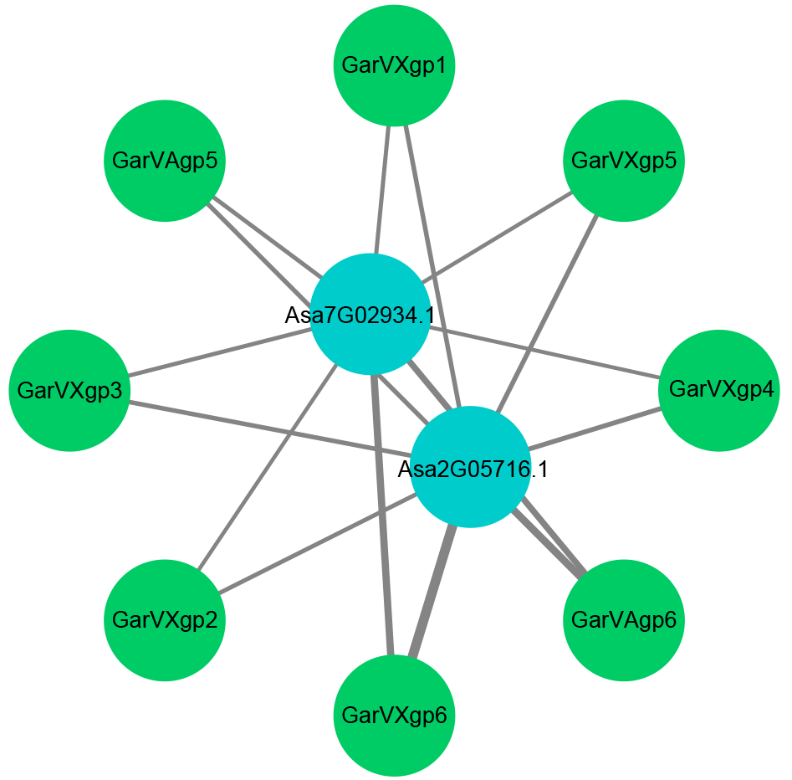


**Fig. S13:** Co-expression network of garlic heat stress protein-encoding genes and virus genes. *GarVXgp1*, *GarVXgp2*, *GarVXgp3*, *GarVXgp4*, *GarVXgp5*, *GarVXgp1*, are genes of Garlic virus X; *GarVAgp5* and *GarVAgp6* are genes of Garlic virus A. The thickness of line between two genes indicates the co-expression level.


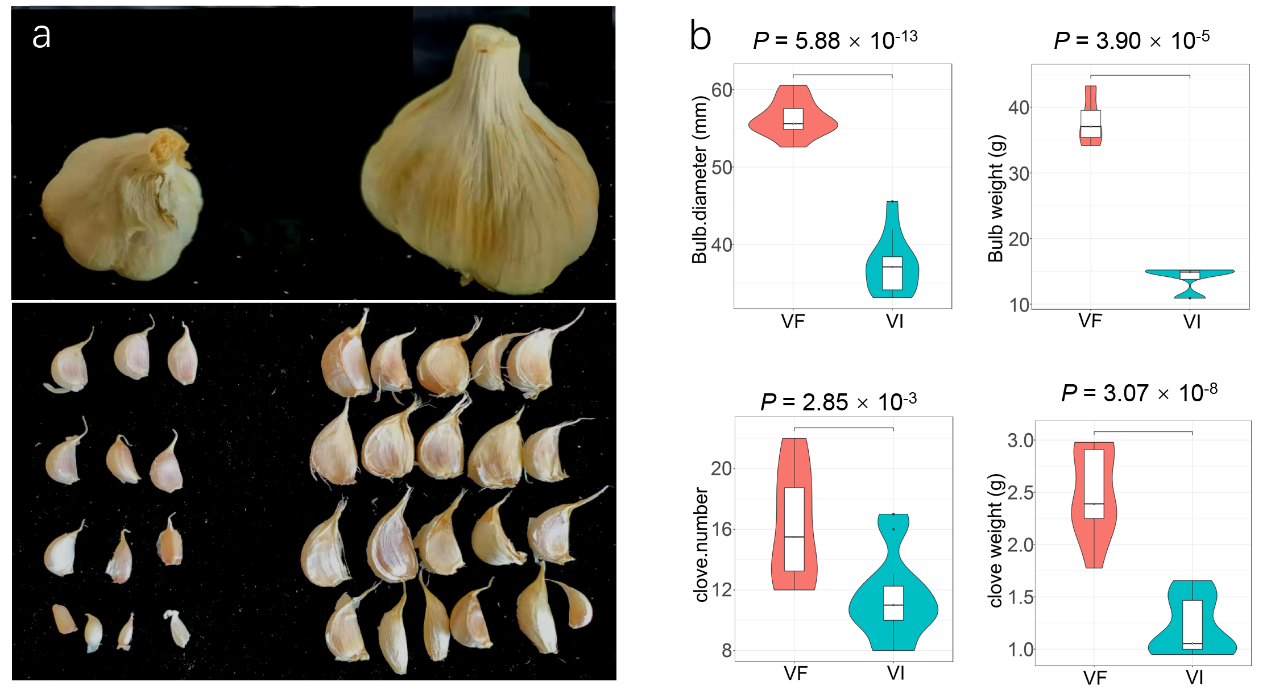


**Fig. S14:** Comparison between bulbs of virus-infected and virus-free garlic. (**a**), The figure shows bulb size and clove number of virus-infected (left) and virus-free (right) garlic. (**b**), Violin plots show the differences for four bulb traits between virus-infected (VI) and virus-free (VF) garlic.


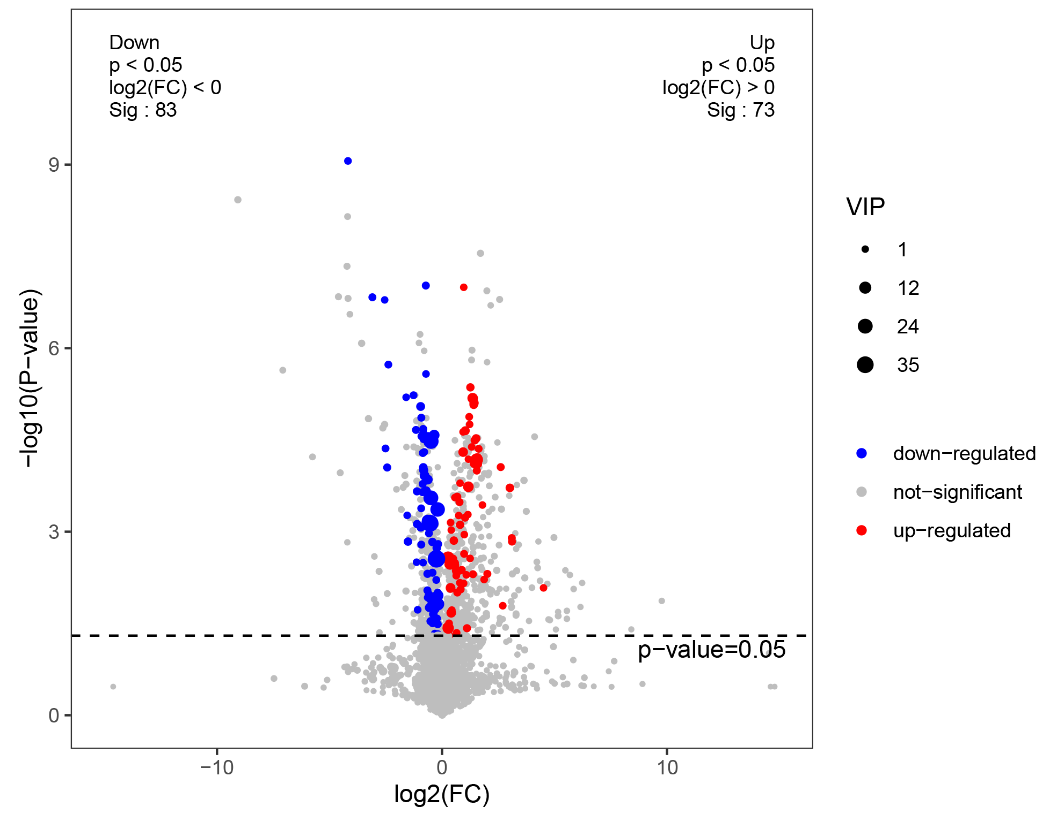


**Fig. S15:** Differential metabolites in the bulb of virus-invasive garlic compared to that of virus-free garlic, from the LC-MS-based untargeted metabolomic analysis.


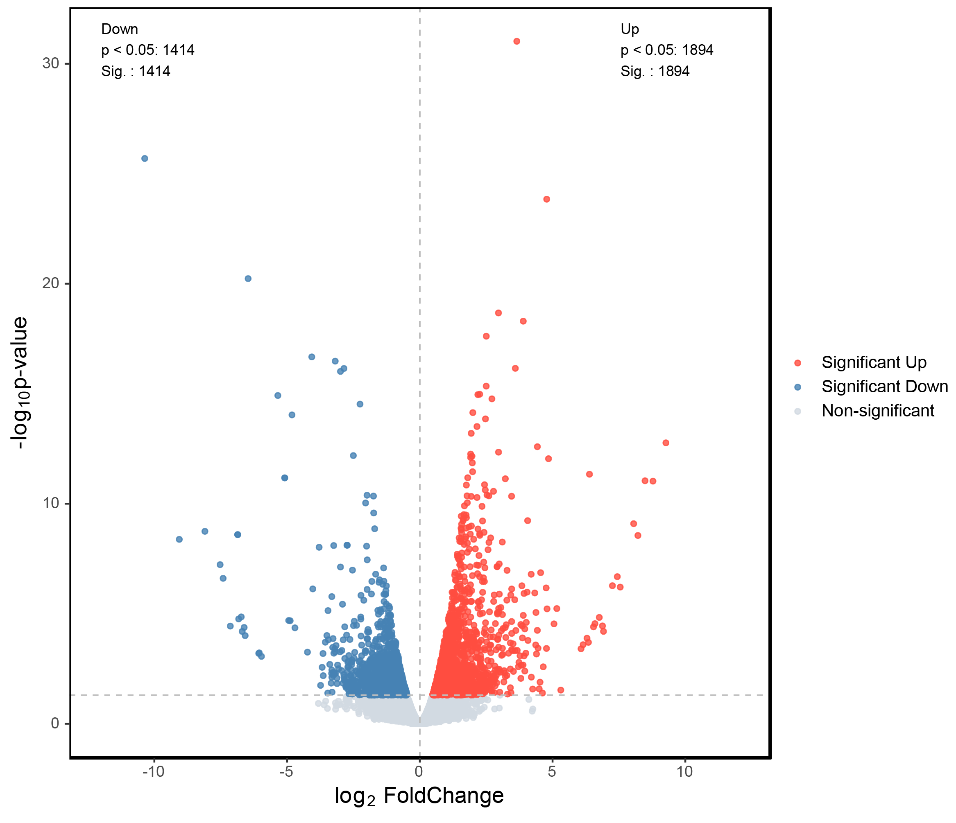


**Fig. S16** Differentially expressed genes in the bulb of virus-invasive garlic compared to that of virus-free garlic.


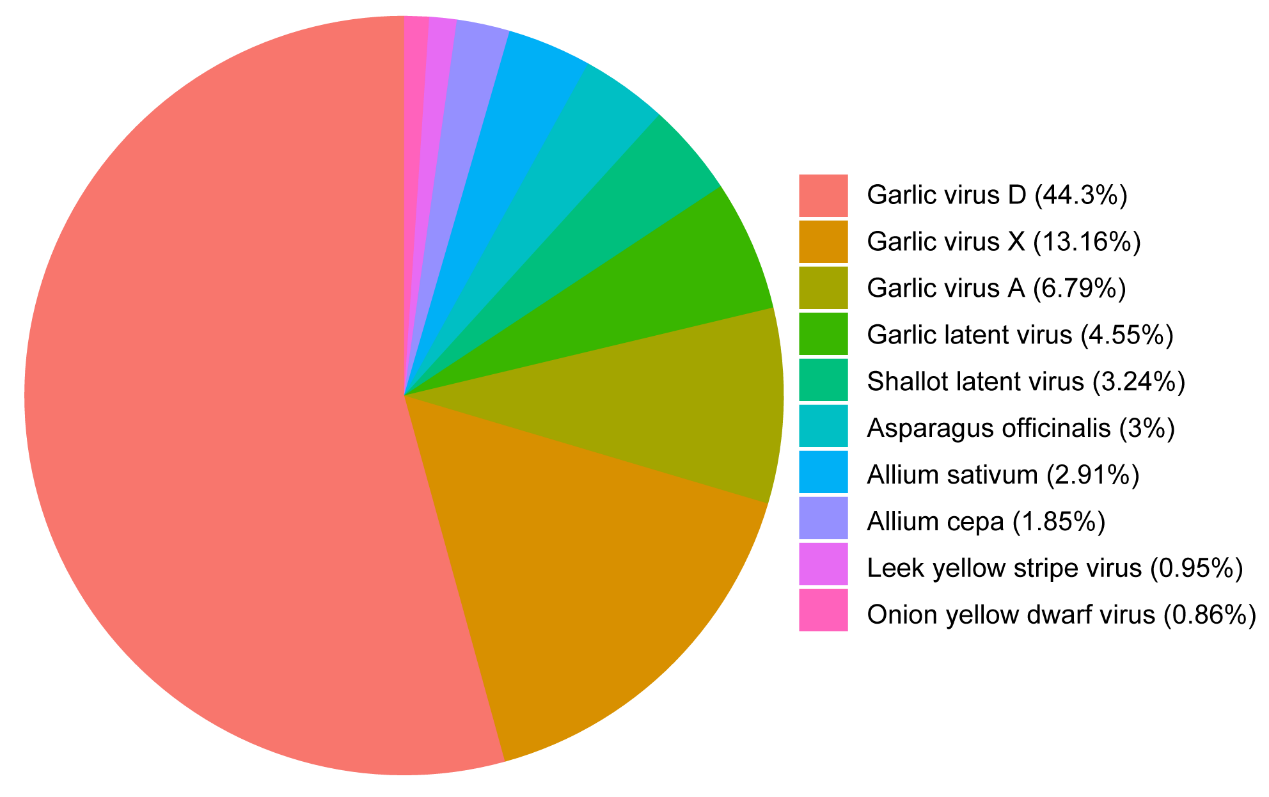


**Fig. S17** Source distribution for the reads unaligned onto garlic genome.
