# Supplementary material for: A single-cell transcriptomic atlas reveals the cell differentiation trajectory and the response to virus invasion in swelling clove of garlic
Source: Hortic Res. 2025 Jan 3;12(4):uhae365. doi: 10.1093/hr/uhae365 (PMC11894531; doi:10.1093/hr/uhae365)
Supplement: Web_Material_uhae365 [file web_material_uhae365.zip › Supplementary Table.docx]

**Table S1** Statistics of data from single-cell RNA sequencing

|  | Bulb-1 | Bulb-2 | Bulb-3 |
| --- | --- | --- | --- |
| Estimated number of cells | 6875 | 6188 | 10568 |
| Cell number after filtering | 6186 | 5281 | 8214 |
| Median genes per cells | 1667 | 1405 | 727 |
| Median UMI counts per cell | 2907 | 2451 | 936 |
| Reads Mapped to Genome of garlic and viruses | 94.6% | 85.0% | 83.5% |
| Percentage of valid barcodes | 96.5% | 93.6% | 94.6% |

**Table S2** Cell number and proportion detected for each cluster in three samples.

|  | bulb-1 |  |  | bulb-2 |  |  | bulb-3 |  |
| --- | --- | --- | --- | --- | --- | --- | --- | --- |
| clusters | Cell number | Frequency (%) |  | Cell number | Frequency (%) |  | Cell number | Frequency (%) |
| C1 | 103 | 1.67 |  | 188 | 3.56 |  | 4839 | 58.91 |
| C2 | 915 | 14.79 |  | 1620 | 30.68 |  | 196 | 2.39 |
| C3 | 1610 | 26.03 |  | 462 | 8.75 |  | 415 | 5.05 |
| C4 | 1225 | 19.80 |  | 914 | 17.31 |  | 339 | 4.13 |
| C5 | 70 | 1.13 |  | 894 | 16.93 |  | 561 | 6.83 |
| C6 | 511 | 8.26 |  | 656 | 12.42 |  | 9 | 0.11 |
| C7 | 898 | 14.52 |  | 147 | 2.78 |  | 97 | 1.18 |
| C8 | 0 | 0.00 |  | 7 | 0.13 |  | 1035 | 12.60 |
| C9 | 1 | 0.02 |  | 166 | 3.14 |  | 713 | 8.68 |
| C10 | 595 | 9.62 |  | 162 | 3.07 |  | 6 | 0.07 |
| C11 | 258 | 4.17 |  | 65 | 1.23 |  | 4 | 0.05 |

**Table S3** Known meristematic cell-expressed genes and its ortholog of garlic

| Known gene | Species | Garlic orthologous gene |
| --- | --- | --- |
| *H3.3_1* | *Oryza sativa* | *Asa8G00577.1* |
| *H3.3_2* | *Oryza sativa* | *Asa3G01236.1* |
| *H2A_1* | *Solanum lycopersicum* | *Asa2G03717.1* |
| *H2A_2* | *Solanum lycopersicum* | *Asa2G03510.1* |
| *H2A_3* | *Solanum lycopersicum* | *Asa5G04088.1* |
| *H2A_4* | *Solanum lycopersicum* | *Asa2G03512.1* |
| *H4* | *Arabidopsis thaliana* | *Asa0G00244.1* |
| *H3* | *Arabidopsis thaliana* | *Asa8G00551.1* |

**Table S4** Average expression level of virus genes in investigated cloves

| Gene | Virus | bulb-1 | bulb-2 | bulb-3 |
| --- | --- | --- | --- | --- |
| *GarVAgp1* | Garlic virus A | 0.0017 | 0.0359 | 0.0429 |
| *GarVAgp2* | Garlic virus A | 0 | 0.0124 | 0 |
| *GarVAgp3* | Garlic virus A | 0 | 0.0021 | 0 |
| *GarVAgp4* | Garlic virus A | 0 | 0.0087 | 0 |
| *GarVAgp5* | Garlic virus A | 0 | 0.0528 | 0.0008 |
| *GarVAgp6* | Garlic virus A | 0.0055 | 0.3825 | 0.0001 |
| *X660-gp1* | Garlic virus D | 0.0003 | 0.0054 | 0.0249 |
| *X660-gp2* | Garlic virus D | 0 | 0.0007 | 0.0118 |
| *X660-gp3* | Garlic virus D | 0.0002 | 0.0022 | 0.0096 |
| *X660-gp4* | Garlic virus D | 0.0006 | 0.0022 | 0.0441 |
| *X660-gp5* | Garlic virus D | 0.0023 | 0.0238 | 0.1707 |
| *X660-gp6* | Garlic virus D | 0.0146 | 0.3191 | 1.7177 |
| *GarVXgp1* | Garlic virus X | 0 | 0.0266 | 0 |
| *GarVXgp2* | Garlic virus X | 0 | 0.0119 | 0 |
| *GarVXgp3* | Garlic virus X | 0 | 0.0224 | 0 |
| *GarVXgp4* | Garlic virus X | 0 | 0.0247 | 0 |
| *GarVXgp5* | Garlic virus X | 0.0004 | 0.0485 | 0 |
| *GarVXgp6* | Garlic virus X | 0.0116 | 0.9338 | 0 |
| *GarLVgp1* | Shallot latent virus | 0.0004 | 0.0159 | 0.5816 |
| *GarLVgp2* | Shallot latent virus | 0 | 0.0054 | 0.0318 |
| *GarLVgp3* | Shallot latent virus | 0 | 0 | 0 |
| *GarLVgp4* | Shallot latent virus | 0 | 0.0003 | 0.0009 |
| *GarLVgp5* | Shallot latent virus | 0.0001 | 0.0087 | 0.0400 |
| *GarLVgp6* | Shallot latent virus | 0.0004 | 0.0584 | 0.4107 |
| *QY65-gp1* | Garlic virus B | 0.0003 | 0.0514 | 0.1956 |
| *QY65-gp2* | Garlic virus B | 0 | 0.0065 | 0.0118 |
| *QY65-gp3* | Garlic virus B | 0 | 0.0040 | 0.0195 |
| *QY65-gp4* | Garlic virus B | 0 | 0.0011 | 0.0142 |
| *QY65-gp5* | Garlic virus B | 0.0002 | 0.0512 | 0.2826 |
| *QY65-gp6* | Garlic virus B | 0.0033 | 0.5272 | 2.4033 |
| *GvEgp1* | Garlic virus E | 0.0007 | 0.0361 | 0.4895 |
| *GvEgp2* | Garlic virus E | 0 | 0.0006 | 0.0358 |
| *GvEgp3* | Garlic virus E | 0 | 0.0014 | 0.0116 |
| *GvEgp4* | Garlic virus E | 0.0003 | 0.0013 | 0.0350 |
| *GvEgp5* | Garlic virus E | 0 | 0.0007 | 0.0212 |
| *GvEgp6* | Garlic virus E | 0 | 0 | 0 |
| *GvEgp7* | Garlic virus E | 0 | 0 | 0 |
| *GvEgp8* | Garlic virus E | 0.0018 | 0.3317 | 1.9611 |

**Table S5** Primer sequences used for Real-time fluorescent quantitative PCR

| **Gene** | **Sequence** | |  |
| --- | --- | --- | --- |
| *GarVAgp6* | Forward primer | TACGCCAAAGCATGCTAC | |
|  | Reverse primer | GCGTCGGGATTCAACAAT | |
| *X660.gp6* | Forward primer | CGTGACCCTCCAAATGCT | |
|  | Reverse primer | TAACGAAGCGTGAGGAAC | |
| *GarVXgp6* | Forward primer | CACAGGAGATCGTCCGTG | |
|  | Reverse primer | TGCTTCCCCGTGACATAG | |
| Asa7G05685.1 | Forward primer | AGAGGCTTATACGCCACC | |
|  | Reverse primer | TAGCCTCCATTTCACACCAA | |
| Asa7G01788.1 | Forward primer | ACAACCAAACAGCCGATG | |
|  | Reverse primer | GAAGAAGTCAACTGTAGCCG | |
| Asa1G02974.1 | Forward primer | CAACGTTCGTAGTTTCAACC | |
|  | Reverse primer | ACAACCAAACAGCCGATG | |
| Asa7G06899.1 | Forward primer | GCATCGGCATCTACTACGAC | |
|  | Reverse primer | CCACCTCAATTTCCCTTGCA | |
| Asa2G06168.1 | Forward primer | ATCTTTGTATTCGCCGCAGA | |
|  | Reverse primer | TAGCAAACAGGCGACAACC | |
| Asa3G03655.1 | Forward primer | GACATGTACAGCAAAGATGG | |
|  | Reverse primer | CAGGAGTTCCGTCAAAAACT | |
